# Environmental selection underlies distinct distribution patterns of closely related European evening primroses

**DOI:** 10.1038/s41598-025-88888-3

**Published:** 2025-02-05

**Authors:** Monika Woźniak-Chodacka, Maciej Kocurek, Maria Pilarska, Ewa Niewiadomska

**Affiliations:** 1 W. Szafer Institute of Botany, Polish Academy of Sciences, Lubicz 46, 31-512 Kraków, Poland; 2 Institute of Biology, The Jan Kochanowski University, Uniwersytecka 7, 25-406 Kielce, Poland; 3 The Franciszek Górski Institute of Plant Physiology, Polish Academy of Sciences, Niezapominajek 21, 30-239 Krakow, Poland

**Keywords:** Species distribution, Ecological selection, Europe, Evening primrose, *Oenothera*, Photosynthesis, Gas exchange, Ecology, Evolution, Physiology, Plant sciences

## Abstract

Understanding of species distribution is becoming a key concern in biogeography, ecology and evolution when to consider the ongoing climate change. This study investigates the distribution patterns of closely related *Oenothera* species focusing on their adaptations to environmental conditions through morphological traits and photosynthetic adjustments. We documented the three distinct distribution patterns in Europe among the studied species. *Oenothera rubricaulis*, found at higher latitudes, has the smallest flowers and the highest chlorophyll content, accompanied by anthocyanin accumulation, which maximizes light acquisition under low light conditions. *Oenothera suaveolens*, observed at lower latitudes, displays the largest flowers, a pure green phenotype, the highest stomatal conductance, and a light avoidance strategy, reflected by rapid photoinhibition and hyponasty. *Oenothera biennis*, with the widest distribution, exhibits an intermediate phenotype, suggesting high plasticity and adaptability of its photosynthetic apparatus. Given the close relationship of these species, our findings suggest that environmental selection following hybridization events has been crucial for their establishment in Europe.

**Supplementary Information:**

The online version contains supplementary material available at 10.1038/s41598-025-88888-3.

## Introduction

Among several factors affecting plant distribution, the most profound effect on natural distribution has climate^1,2^ whereas distribution of non-native species is also strongly influenced by the history of introduction^3,4^. By adapting to the new or changing surroundings, plants increase their chances of survival and reproduction in their ecosystem and, consequently, the possibility of further spreading. Understanding species distribution and their ability to adapt is particularly important in the face of ongoing climate change.

In order to succeed in their environment, plants need to adjust their morphological and physiological features. Adjustments can refer to short-term responses, long-term acclimatization and fixed, heritable adaptations^5,6^. Apparently, the most important climatic stimuli regulating plant growth and development are light and temperature. In regard to temperature, some common phenotypic effects on plant architecture were demonstrated with *Arabidopsis thaliana* rosette^7^. Plants grown at an optimal temperature (22 °C) formed a big rosette composed of large leaves. At low temperature (16 °C) they formed dwarf and compact rosette with small leaves. At high temperatures (28 °C), however, they formed small leaves with elongated petioles, often accompanied by leaf hyponasty. As long as mesophyll cells are considered, the role of climatic sensors might be attributed to chloroplasts because they make a direct use of sunlight to transform it into the metabolically usable energy of chemical bonds. Chloroplasts are also capable of using this energy for assimilation of CO_2_ into the carbon skeletons of organic matter in the process of photosynthesis. Hence, they represent a cellular hub performing and integrating a wide range of physiological responses resulting from abiotic environmental changes^8^.

Photosynthesis, due to its considerable variability within and among species, is also a highly adaptive process and a trait under selection in changing environments^9,10^. Plants grown at variable light conditions respond to light differently. This can be visualized by the light curves of photosynthesis. Plants acclimated to higher irradiance activate the photosynthesis faster and are less pronounced to photoinhibition^11^. Athough the genus *Oenothera* has appeared in scientific literature for more than a century, as detailed in the works of Burnham^12^, Cleland^13^, and Harte^14^, its photosynthesis has only been addressed recently^10^. Furthermore, genus *Oenothera* sect. *Oenothera* serves as a valuable model to study plant adaptations. Evening primroses exhibit a wide range of genetic diversity and, as a result, they possess significant phenotypic plasticity. This enables them to adapt their morphology and physiology in response to fluctuating environmental conditions. Moreover, several *Oenothera* species have a relatively short generation time and can undergo rapid evolutionary changes, making them well-suited for experimental studies of adaptation. Noteworthy, some species, such as *Oenothera biennis*, are economically important crop plants for their seeds containing essential fatty acids (mainly gamma-linolenic acid [GLA]) broadly used in pharmacy, medicine and in the production of biodiesel^15,16^. Studying the adaptations of these species can have practical applications in agriculture, biotechnology, and medicine.

Previous study on *Oenothera* adaptations were focused mostly on American collective species and experimentally created hybrid lineages, often displaying an incompatibility between the chloroplast and the nuclear genomes (plastome-genome incompatibility [PGI])^17–19^. For instance, it was recently demonstrated that evening primroses with distinct plastome-genome arrangements exhibit notable variations in their response to contrasting light conditions. Specifically, *Oenothera elata*, characterized by the AA-I plastome-genome combination and indigenous to the Western United States and Mexico, demonstrates superior adaptability to high light intensities compared to species possessing the AB-II arrangement, such as *Oenothera biennis*, which is native to Eastern American woodland habitats^10,20^.

**Fig. 1:**
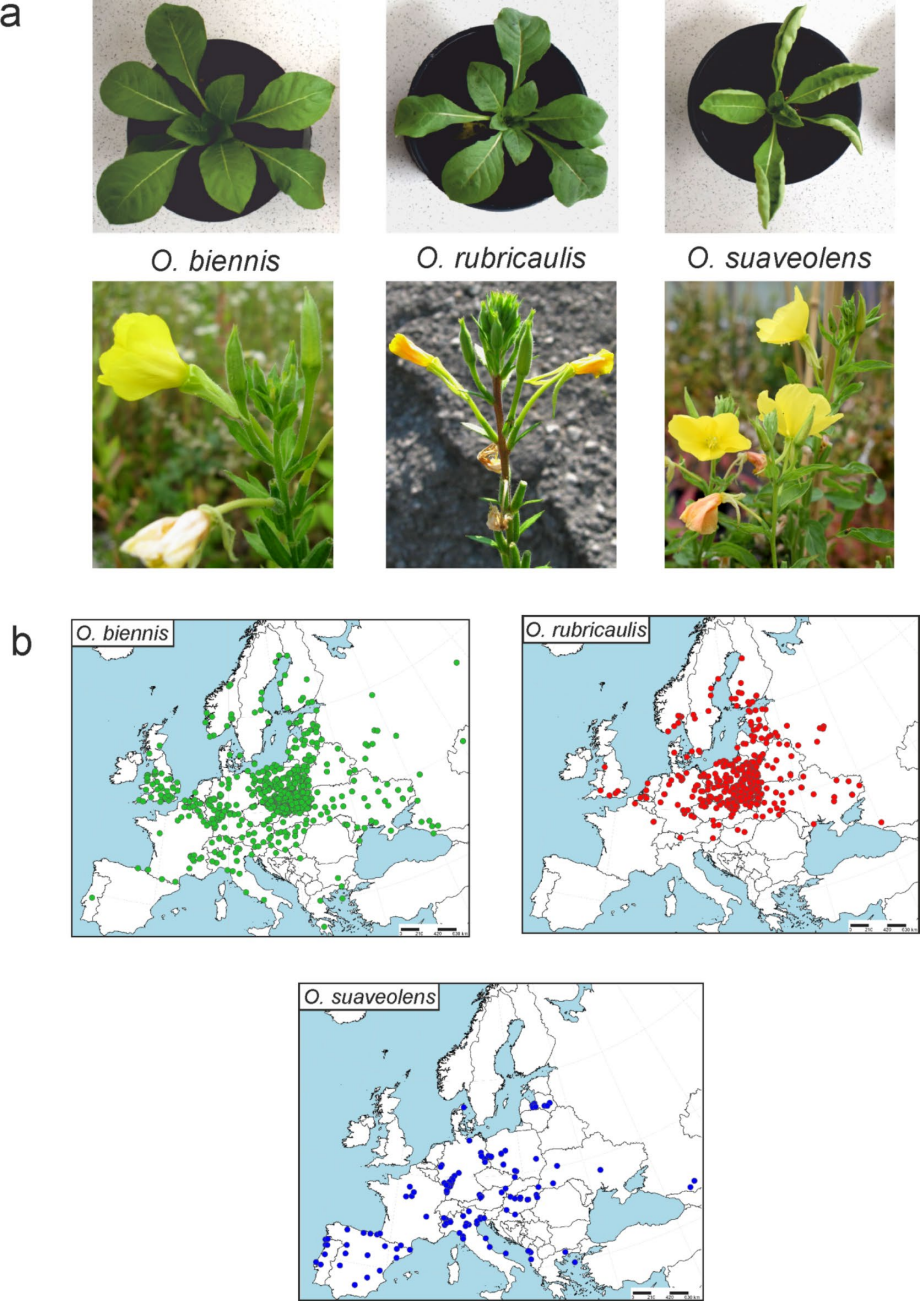
The morphology (**a**) of the three species at the stage of an early rosette (6–8 weeks) and during the flowering phase and their European distribution (**b**) based on the herbal collections and original data.

Less is known about European evening primroses. It is assumed that many *Oenothera* species were introduced into the Old World during the last 350 years. In Europe, they found favourable conditions and started to spread and hybridize with each other^13^. To study morphological and physiological adaptations, we selected three European *Oenothera* species: *O. biennis*, *O. rubricaulis*, and *O. suaveolens* (Fig. 1a). The species are relatively widely distributed and form distinct, although partially over-lapping ranges in Europe. However, no detailed maps are available since data on the distribution of particular species are scattered through the literature. Moreover, the species are very closely related as they share their haploid genomes: *O.biennis* shares one of its haploid genomes with *O. rubricaulis* and the other one with *O. suaveolens*. On this basis, *O. biennis* it thought to be parental species for both *O. rubricaulis* and *O. suaveolens*^13^.

The aim of the research was to elucidate patterns of distribution of closely related European *Oenothera* species in terms of their adaptation to environmental conditions, manifested by adjustments of photosynthesis parameters. The specific goals were: (1) to unravel detailed patterns of distribution of the chosen European evening primrose species; (2) to explore a morphological and physiological variation of the studied taxa in the context of their adaptation to local environments; and (3) to investigate whether physiological profiles reflected by photosynthetic parameters can be associated with the distribution of the species.

## Results

### Distribution of the three species in Europe

The data on localization of the studied species were used to prepare the detailed maps of distribution (Fig. 1b). As can be seen, *Oenothera biennis* occupies greatly extended ranges across the whole continent. The species is abundant, especially in Central and East Europe and in the United Kingdom; the stands from southern regions of the continent are less numerous. Nevertheless, the southern limit of distribution reaches 37° Northern latitude in Peloponnese (Greece)^21^ and the northern limit of distribution reaches 65° Northern latitude, along the shore of the Baltic Sea in Piteå and Nederkalix (Sweden) and Kemi (Finland)^22^. The species spreads from Abrantes in Portugal (8° Western longitude)^23^ to 40° Eastern longitude where several localities were noted from Causas (e.g. Nikiel), a region localized between two continents, Europe and Asia^24^.

The distribution of *O. rubricaulis* is much more limited. The species grows mainly in Central, East and North parts of Europe and is locally abundant. Similarly, as in the case of *O. biennis*, the northern limit reaches 65° Northern latitude in Oulu (Finland)^22^, but the southern limit reaches only 44° Northern latitude in the territory of Russia (e.g. Nikiel) (KTU). The western boundary of distribution is localized at 3° Western longitude in Somerset County and Lancashire County in Great Britain^25^, and the eastern limit extends 37° Eastern longitude in Moscow (Russia) (KTU).

On the contrary, *O. suaveolens* was observed mainly in the southern parts of the continent. The northernmost locality of the species was noted from one stand in Denmark, where the species reaches 57° Northern latitude^22^. In the South it extends to 37.8° Northern latitude in Jaen in Spain^26^. In the West, the species was observed as far as 28° Western longitude, on Faial Island, which is a Portuguese Island of the Central Group of the Azores (KTU). In Continental Europe, the species spreads from 8° Western longitude in Portugal to 48° Eastern longitude, where it was noted from Mozdoskij in Russia.

If to generalize the localization of the three species in Europe, *O. rubricaulis* seems to be adapted to the higher latitudes and *O. suaveolens* to the lower ones. Whereas, *O. biennis* has the most extended range of occurrence.

### Morphological differentiation of the three species of Oenothera

**Fig. 2:**
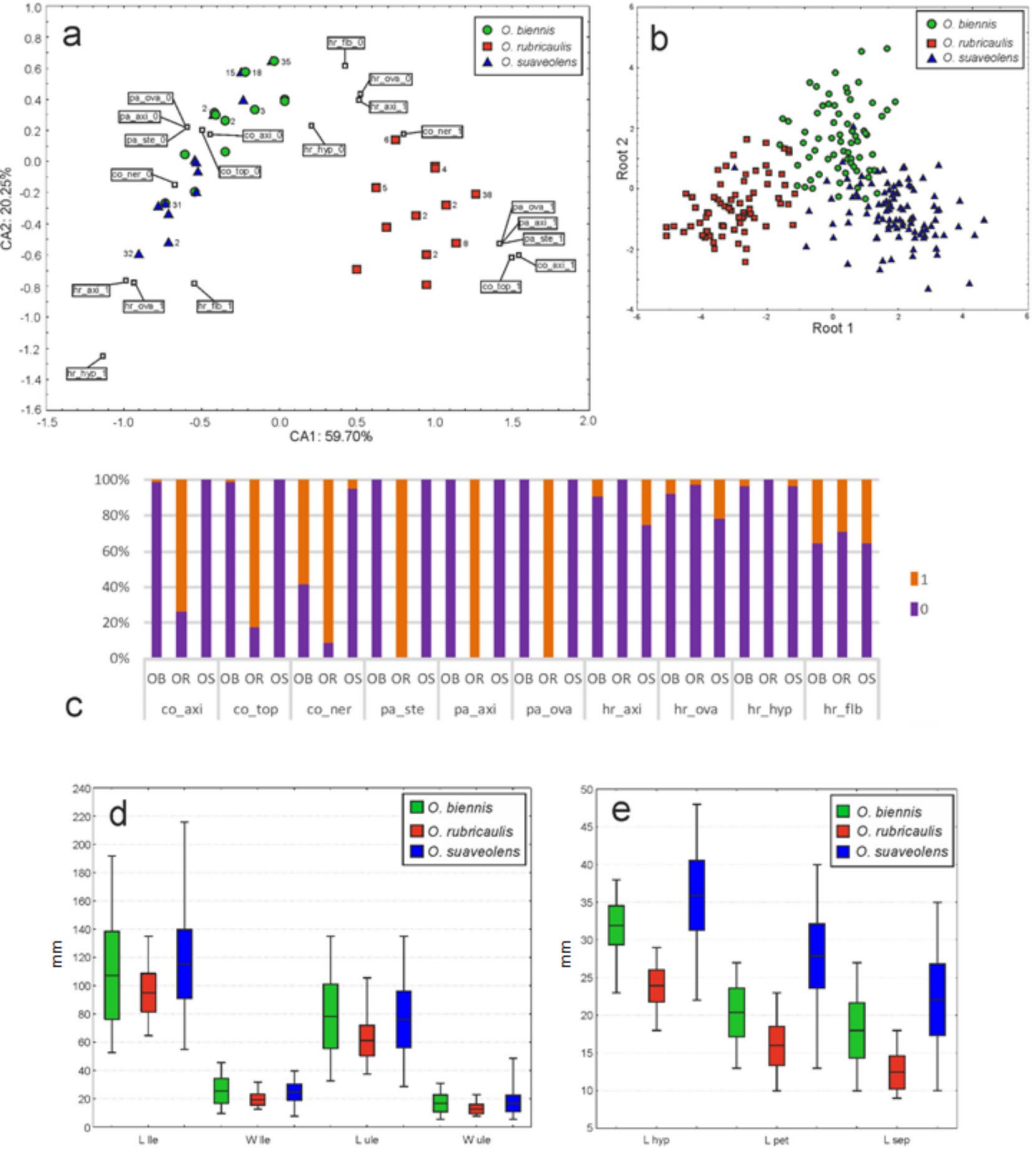
Morphological differentiation of the three *Oenothera* species based on qualitative and quantitative features (list of features is presented on Table 1): (**a**) Two-dimensional ordination diagram of correspondence analysis (CA) along CA1 and CA2. The numbers indicate the number of specimens to which a particular point refers to (given only if more than one). Black empty squares show the location of the defined states of the features: co_axi_gr/rd – inflorescence axis colour green/ red; co_top_gr/rd – top of the inflorescence axis colour green/ red; co_ner_gr/rd – main nerves colour green/ red; pa_ste_gr/rd – papillae on the inflorescence axis colour green/ red; pa_ova_gr/rd – papillae on the ovaries colour green/ red; hr_axi_gla/str – predominant hair type on inflorescence axis; hr_ova_gla/str – predominant hair type on ovaries; hr_hyp_gla/str – predominant hair type on hypanthia; hr_lfb_gla/str – predominant hair type on flower buds. (**b**) A scatter diagram showing results of canonical discriminant analysis (CDA) of all quantitative data. Each point represents one specimen. (**c**) The frequency of the defined states (“0” and “1”) of the studied qualitative features. The state “0” refers to green, while “1” refers to red colour of particular organs (inflorescence axis, top of the inflorescence axis, nerves, papillae on the stem, inflorescence axis and ovaries). It also refers to the predominance of glandular (“0”) vs. strigillose (“1”) hairs on the selected plant structures (inflorescence axis, ovaries, hypanthia, and flower buds). (**d**) The variability range of the chosen vegetative features: L_lle [lower leaves length], W_lle [lower leaves width], L_ule [upper leaves length] and W_ule [upper leaves width]. (**e**) The variability range of the chosen generative features: L_hyp [hypanthium length], L_pet [petals length], and L_sep [sepals length]. The lines within the boxes indicate the mean values, the boxes represent the mean value +/ - standard deviation, and the whiskers around the boxes refer to the min/max values among the species, respectively.

The relationships between the qualitative characteristics of the three species were analyzed by correspondence analysis (CA). The resulting two-dimensional plot (Fig. 2a) demonstrates a grouping referring to *O. rubricaulis* individuals and two partially overlapping sets of points representing specimens belonging to *O. biennis* and *O. suaveolens*. *Oenothera rubricaulis* turned out to be the most distant one since it contains considerable amounts of red anthocyanins in the tissues^25^, whereas the two remaining species are in general pure-green, except for main nerves in some representatives of *O. biennis*. As the research show, representatives of *O. biennis* are in general covered mostly by glandular hair, and individuals of *O. suaveolens* have less glandular and more strigillose hair. Moreover, the analysis of the frequencies of the defined states of qualitative characteristics shown the general invariability of the species in the whole ranges of distribution (Fig. 2c).

**Table 1:** The list of examined characters and their abbreviations.

No.	Description	Abbr.	Scale/ unit/ category
Qualitative features			
1	Inflorescence axis colour	co_axi	0 = green [gr], 1 = red [rd]
2	Top of the inflorescence axis colour	co_top	
3	Main nerves colour	co_ner	
4	Papillae on the stem colour	pa_ste	
5	Papillae on the inflorescence axis colour	pa_axi	
6	Papillae on ovaries colour	pa_ova	
7	Predominant hair type on inflorescence axis	hr_axi	0 = glandular [gla], 1 = strigillose [str]
8	Predominant hair type on ovaries	hr_ova	
9	Predominant hair type on hypanthia	hr_hyp	
10	Predominant hair type on flower buds	hr_flb	
Quantitative features & ratios			
11	Lower leaves length	L_lle	mm
12	Lower leaves width	W_lle	
13	Upper leaves length	L_ule	
14	Upper leaves width	W_ule	
15	Bracts length	L_bra	
16	Bracts width	W_bra	
17	Hypanthium length	L_hyp	
18	Petals length	L_pet	
19	Filaments length	L_fil	
20	Anthers length	L_ant	
21	Style length	L_sty	
22	Stigma length	L_sti	
23	Ovary length	L_ova	
24	Sepals length	L_sep	
25	Sepal tips length	L_spt	
26–34	L/W_lle, L/W_ule, L/W_bra, L_ant/fil, L_sti/sty, L_sty/fil, L_sty/pet, L_pet/hyp, L_spt/sep		ratio

Quantitative traits were subjected to the principal component analysis (PCA). The scatterplot according to the first two components (supplementary Fig. S1) showed strongly overlapping gatherings referring to particular taxa. The most distant from each other along the first axis were groups composed of individuals of *O. suaveolens* and *O. rubricaulis*. Next, discriminant analysis (DA) followed by canonical discriminant analysis (CDA), was carried out. Detailed results of these approaches are presented in supplementary Table S1. As shown in Fig. 2b, the three groups of individuals refer to separate and well-defined species. According to the quantitative features, the species can be easily distinguished mainly by the size of flower elements (Fig. 2d, e); for more details see supplementary Table S1. In summary, analyses of the chosen morphological features lead to the conclusion that *O. rubricaulis* and *O. suaveolens* are the most distant, whereas *O. biennis* is placed somewhere in between these two species.

### Comparison of photosynthetic parameters of the three species

**Fig. 3:**
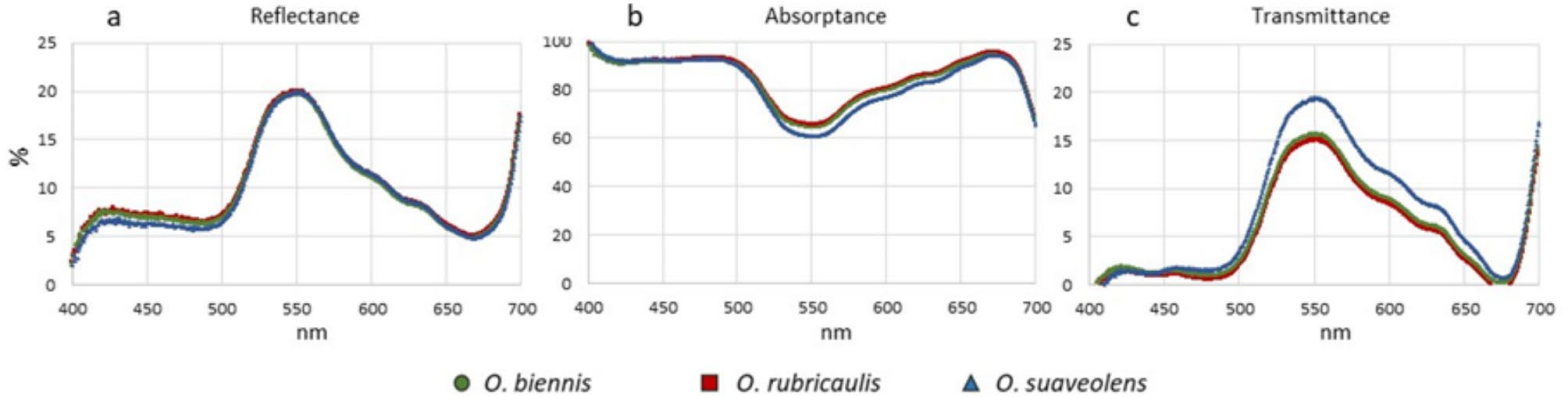
Leaf optical properties: reflectance (**a**), absorptance (**b**) and transmittance (**c**), determined in leaves of the three *Oenothera* species. Each line represents the averaged curve (*n* = 5).

To get an insight into the structure of leaves we determined their optical properties (Fig. 3). Reflectance, a characteristic referring to the fraction of light reflected by the leaf surface, was similar in the three species (Fig. 3a), except for slightly lower value for *O. suaveolens* in blue light, 420–500 nm wavelength, in which only about 6‒7% of flux was reflected. The highest percentage of scatter light was observed in 530–570 nm (green-yellow light); at longer wavelengths the value decreased rapidly. The second peak was localized in infrared region of the spectrum. Conversely, absorptance which refers to the portion of light absorbed by a leaf blade, produced high and fairly constant values (90% and higher) in blue light region (400–500 nm) for all three species (Fig. 3b). The lowest absorptance was observed in 550 nm, especially in *O. suaveolens* leaves (60% vs. 65% in *O. biennis* and *O. rubricaulis*). The tendency for the curve of *O. suaveolens* to produce lower values comparing to the two remaining species was most prominent in 500–670 nm range. Transmittance is the fraction of light passing the leaf. Transmittance curves of *O. biennis* and *O. rubricaulis* showed minimal differences along almost the whole spectrum, whereas the curve of *O. suaveolens* was apparently different (Fig. 3c). The maximum values were noted in 550 nm, 14%, 16% and 19% for *O. rubricaulis*, *O biennis* and *O. suaveolens*, respectively. In 400–500 nm transmittance showed minimal values of 2‒3%. Transmittance rapidly dropped with increasing wavelength (560 nm and longer) to a minimum value in 670–680 nm, with a second peak in an infrared region.

**Fig. 4:**
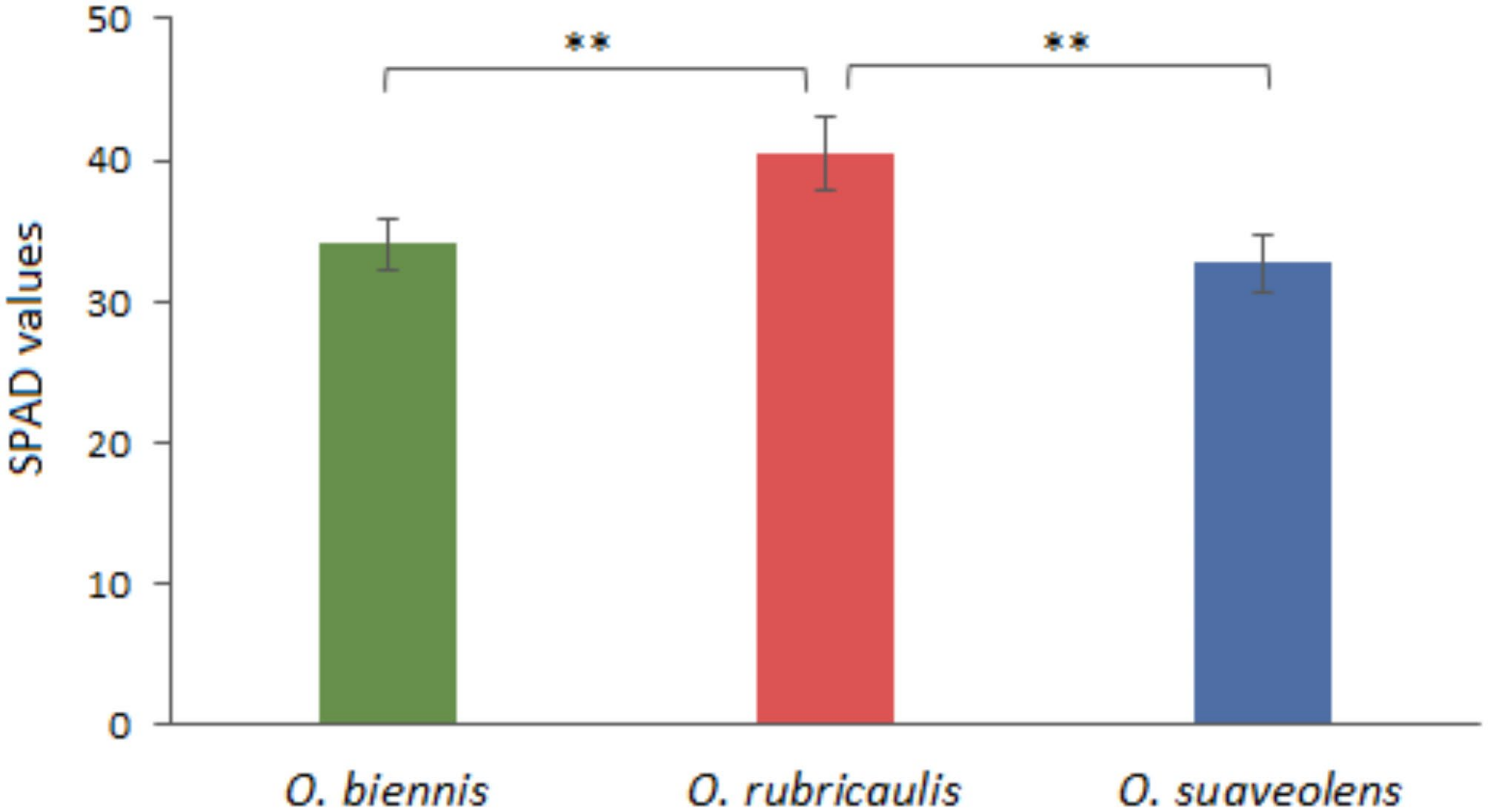
SPAD values measured on leaves of the three *Oenothera* species. Data represent mean ± SD (*n* = 10).

In the studied taxa, the SPAD values which are correlated with chlorophyll content were significantly higher *in O. rubricaulis* (value of 40) in comparison to the two remaining species, and amounted to the values of 34 in *O. biennis* and 32 in *O. suaveolens* (Fig. 4).

**Fig. 5:**
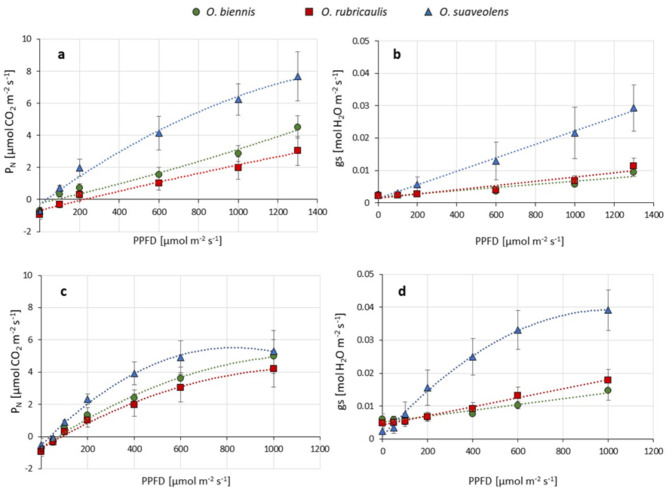
Net photosynthetic rate (Pn) (**a**, **c**) and stomatal conductance (gs) (**b**, **d**) measured on leaves of the three *Oenothera* species in red (a-b) and blue (c-d) actinic light. Each point represents mean ± SD (*n* = 4).

The action spectra of photosynthesis have two peaks (in blue and in red light range) but typically net photosynthesis (Pn) is measured under red light because, in comparison to blue light, it is less harmful for photosynthetic antenna and penetrates deeper into the leaf. Pn rates measured in red light were outstanding high in *O. suaveolens* (almost 8 µmol CO_2_ m^2^ s^− 1^ at 1300 µmol PPFD m^2^ s^− 1^) in comparison to the other two species. However, the slowest Pn rates were measured in *O. rubricaulis* (3 µmol CO_2_ m^2^ s^− 1^ at 1300 µmol PPFD m^2^ s^− 1^) (Fig. 5a). The highest rates of Pn in *O. suaveolens* were accompanied by the highest values of stomatal conductance (gs) (0.03 at 1300 µmol PPFD m^2^ s^− 1^) (Fig. 5b). While *O. biennis* and *O. rubricaulis* were quite similar in this respect (about 0.01 at 1300 µmol PPFD m^2^ s^− 1^). Hence, it is reasonable to assume that intensive CO_2_ acquisition in *O. suaveolens* may at least in part result from the more open stomata or from their higher density. To verify this ambiguity we checked the density of stomata in abaxial side of leaves (supplementary Fig. S2). This analysis however revealed a similar density of stomata in analyzed species.

Sensitivity to high irradiance was tested with blue light which carries much higher energy than red light and affects predominantly the top mesophyll cells and photosystem II (PSII). Determination of Pn under increasing intensity of blue light revealed a different relationship than that under red and revealed the most rapid saturation of Pn curve in *O. suaveolens* leaves in comparison to the other two species (Fig. 5c). The differences among the species were most apparent in the range of 200‒600 µmol PPFD m^2^ s^− 1^, in 1000 µmol PPFD m^2^ s^− 1^, the results started to become less contrasting and reached 4, 5, and 5 µmol CO_2_ m^2^ s^− 1^ for *O. suaveolens*, *O. biennis* and *O. rubricaulis*, respectively.

Similarly to the picture obtained under red light, under blue light the highest stomatal conductance was also measured in *O. suaveolens* (Fig. 5d). It is well known that blue light typically induce the higher stomatal opening than red light, as intensified by blue light signalling^27^. To quantify this effect we compared the maximal values of gs measured under blue and red and calculated that the strongest blue light-dependent stimulation of gs occur in *O. rubricaulis* (140%), the intermediate stimulation occur in *O. biennis* (128.5%) and the lowest in *O. suaveolens* (102.3%). Taking into consideration the highest gs values in *O. suaveolens* in red light, a weak stimulation by blue light may suggest that in this species stomata are already near to their maximal limits under red irradiation.

Altogether, this group of data points to the highest efficiency of photosynthesis in *O. suaveolens* despite the relatively low chlorophyll content (represented by SPAD values) and the greatest stomatal opening.

## Discussion

### Mechanisms affecting distribution of evening primroses in Europe

To gain insight into the adaptation potential of the three *Oenothera* species, we analyzed their distribution in Europe. Despite the efforts made, none of these species were found in America^13,28^. Although most of the Europe lies within the temperate climate zone, it encompasses a considerable variation of climatic conditions. The temperate climate area can be further divided into warm, transitional, and cool zones depending on the average temperatures, as well as to Atlantic, transitional, and continental climates based on mean precipitation values^29^. Additionally, the southernmost regions of the continent, including the Iberian Peninsula, the Apennine Peninsula, and the Balkan Peninsula, are affected by the Mediterranean climate. In contrast, the northernmost region, comprising the Scandinavian Peninsula and the Kola Peninsula, is influenced by arctic and subarctic climates.

The obtained distribution maps revealed distinct patterns of occurrence for the three species. *Oenothera biennis*, which grows over a wide range of latitudes, is present in all temperate climates, from the temperate cool Atlantic zone in Northwest Europe to the temperate warm continental zone in southwest part of the continent. Moreover, the species also spreads through all three peninsulas mentioned above, which are influenced by the Mediterranean climate. This likely indicates high plasticity and adaptability of the species. In contrast, *Oenothera rubricaulis* has successfully colonized only those parts of the continent influenced by temperate climates. Similar to *O. biennis*, *O. rubricaulis* grows along the coasts of Scandinavian countries and further spreads through Eastern and Central Europe. However, the species has failed to colonize the hottest regions of the continent. Conversely, *Oenothera suaveolens* grows mostly in Southern and Central Europe, reaching as far north as Denmark. Thus, it occupies areas influenced by Mediterranean and temperate warm climates but seems incapable of populating Scandinavia. Additionally, it has not been noted in the British Isles.

The distribution of several elements of European vegetation is typically analyzed in the context of glacial and interglacial periods during the Pleistocene. The possible consequences of these cyclic climatic changes include extinction, survival in refugia, postglacial migrations, and more. These mechanisms undoubtedly had a profound effect on American *Oenothera* species. However, the distribution of evening primroses in Europe has been determined by different mechanisms, as a significant number of these plants were introduced to Europe long after the last glacial period^13^.

The Industrial Revolution had a profound effect on the distribution of evening primroses due to the significant improvement in transport infrastructure, characterized by networks of roads, railways, waterways, and canals. In addition, many forests have been cut down, partly to provide lumber and partly to be transformed into urban areas for a rapidly growing human population. The expansion of favourable habitats such as roadsides, railway embankments, and wastelands provided the evening primroses with opportunities for successful colonization of the European continent.

Lastly, it is noteworthy that the predominant type of pollination within the genus *Oenothera* is self-pollination^13^. Although flowers opening at sunset can be accidentally pollinated by moths, potentially leading to pollen exchange between species (i.e., hybridization), most flowers are usually self-pollinated before opening. This fact implies that the distribution of particular evening primrose species is independent of the distribution of any specialized or general pollinator.

### Variability of morphological traits in the context of adaptation

Besides genes, the environment is the second most powerful factor shaping plants’ phenotype. Climate, local conditions, the presence of herbivores, symbiotic microorganisms, and many other factors affect the appearance of plants. Plants growing along large elevational or latitudinal ranges can adapt to local environments. For instance, plants adapted to severe climates in northern or mountainous regions usually present a more compact habit and have smaller flowers compared to individuals growing under milder conditions. Environmental gradients can cause intraspecific variability, such as within *Bellidiastrum michelii*^30^, as well as species replacement, where closely related species replace each other depending on local conditions, e.g., *Rhododendron hirsutum* and *R. ferrungineum*^31^.

In the present study, no signs of intraspecific diversity associated with adjustment to local environments were detected (supplementary Figs. S3 and S4). On the contrary, each species has shown a highly uniform appearance, despite the latitude and local conditions in which particular individuals grew. This conclusion is consistent with the predominance of self-pollination and the strong suppression of recombination processes within the genus.

Nevertheless, significant differences among the studied species were revealed, both in quantitative and qualitative features. *Oenothera rubricaulis* exhibited the smallest flowers and the shortest hypanthia (the elongated part of receptacle where the style is located). A short hypanthium likely reduces the time required for pollination, as the pollen tube can more swiftly reach ovaries containing ovules. This adaptation may reflect the species’ response to the short vegetation periods in the northern regions of the continent. Additionally, the relatively small and less attractive flowers to potential pollinators may result from a reduction of perianth, accompanying the evolutionary trend towards self-pollination observed in many *Oenothera* species. In contrast, *Oenothera suaveolens*, predominantly found in low-latitude areas, exhibited the largest flowers and the longest hypanthia among the taxa studied. This suggests that the Mediterranean climate, characterized by a longer vegetation period and higher temperatures, does not impose a selective pressure for flower size reduction. Moreover, the fragrant flowers are more attractive to potential pollinators, potentially increasing the likelihood of hybridization events. In this context, *O. suaveolens* displays a phenotype resembling large-flowered, open-pollinated, ancestral-like *O. grandiflora*, which is native to Florida and adjacent areas in America^20^.

It has been shown that *Oenothera rubricaulis* is the most distinguishable among the species due to the red pigmentation of particular organs. Most representatives of this species exhibit a red-colored stem, main nerves, and inflorescence axis (sometimes visible only at the top), as well as red papillae covering the plant. Conversely, *O. biennis* and *O. suaveolens* mostly have pure green phenotypes, despite *O. biennis* displaying colored main nerves. The coloration is primarily dependent on the accumulation of anthocyanins. These compounds have antioxidant properties and may act as a “sunscreen”, protecting the photosynthetic by absorbing the excess energy^32^. Moreover, anthocyanins absorb the green and yellow wavebands of light, between 500 and 600 nm^33^ and therefore improve the employment of scattered solar light, which spectrum is latitude-dependent.

### Physiological profiles reflect evolutionary adaptations

It might be assumed that closely related species have similar leaf properties^34^. However, even within the same species, a great diversification of morphological and physiological traits may be found depending on the origin of the accession. For example, using a plethora of ecotypes of *Arabidopsis thaliana* documented a great variability in chlorophyll content and antioxidant protection^35^. The study presented here compares closely related species of *Oenothera*, revealing several important differences in morphology and in photosynthetic competence that correspond to their adaptations to particular environments.

Firstly, the leaves of the closely related *O. biennis*,* O. rubricaulis*, and *O. suaveolens* differ in their optical properties, as characterized by an increased transmittance in *O. suaveolens*. This characteristic indicates that in the leaves of this species more light reaches the deeper parts of the leaf parenchyma, which might be beneficial for overall photosynthesis. Leaf optical properties (LOPs) depend on both leaf biochemical components, such as chlorophylls and carotenoids, and the mesophyll’s anatomical structure, including the number of cell layers, cell surface area, intercellular volume, and overall leaf thickness^36^. The higher transmittance seems to represent a general strategy of excess light avoidance, which may be achieved in a number of ways, including the: lack of screening pigments in the upper epidermis, low chlorophyll content i.e. the smaller photosynthethic antenna, a strong light-avoiding arrangement of chloroplasts, or other structural changes^37^. LOPs are highly influenced by the light environment of the species. In opposite to that is a lower transmittance of *O. rubricaulis* leaves, accompanied by significantly increased SPAD values indicative for chlorophyll content. A possible explanation for this effect might be the larger antenna of PSII. It is known that the antenna size of photosystem II is regulated by light intensity. In low light the antenna size increases, thus increasing the absorption cross section, but this comes at the cost of a lower PSII efficiency^38^.

Secondly, the three species differ in photosynthetic competences, as shown by the light curves of net photosynthesis. Light curves made with red illumination represent an overall photosynthesis of the leaf because this range of the solar radiation penetrates far into the leaf tissue, hence engages also a deeply located chloroplasts. This is in contrast to the blue region of the solar spectrum, which acts mainly near the leaf surface^39^. The highest rates of Pn under red light were measured in *O. suaveolens*, while those in *O. biennis and O. rubricaulis* were similar. A similar relation between the species was noted for the light curves of stomatal conductance. Therefore, we assume that the highest intensity of photosynthesis in *O. suaveolens* may result, at least in part, from a deeper penetration of light and from a very intensive gas exchange (a better CO_2_ delivery).

Quanta of blue light carry higher energy than quanta of red light, but rates of photosynthesis are typically lower under blue light compared to red^40^. This relation also appears to be true for the *Oenothera* species tested. The reason for this phenomenon is that blue light is used less efficiently by leaves and more rapidly leads to PSII photoinhibition. Light curves of Pn made with blue light resolved that light stress starts earliest in *O. suaveolens*, while in *O. biennis* and *O. rubricaulis* depression of Pn was not yet present at the highest light intensity used. This suggests that the chloroplasts of the upper side of leaves *O. suaveolens* are more pronounced to photoinhibition, in comparison to those in the other two *Oenothera* species. However, it appears that a more rapid photoinhibition of the upper part of the leaf does not negatively affect the overall leaf photosynthesis in *O. suaveolens*. This controversy might be explained by differences in plants architecture. While young plants of *O. biennis* and *O. rubricaulis* form a flat leaf rosette, leaves of *O. suaveolens* are narrow with elongated petioles and exhibit hyponasty. So as, the leaves of *O. suaveolens* seem to realize the strategy of light avoidance as an adaptation to environments where light is in excess.

Pronounced differences between the three species were also related to stomatal conductance. Values of gs measured in blue light were always higher than those measured in red light. This is because blue light-mediated stimulation of stomatal opening is a well-known phenomenon and results from the blue light signaling besides the activation of photosynthesis^41–44^. The highest rates of gs were measured in *O. suaveolens*, whereas in the other two species were similar. However, in all *Oenothera* species tested, the activation of gs by blue light compared to red light was not equal and it was reversibly proportional to the intensity of gs rates under red light. In other words, the weakest stimulation by blue light was noted in *O. suaveolens*. Hence, it might be speculated that in this species the rates of gs were already maximal under red light. As visualized by thermography^27^, the stimulation of gs by the addition of blue to red light enables a better cooling of the leaves. Considering the distribution range of this species in Europe this feature it may be beneficial for cooling in warm climate.

## Conclusions and future perspectives

As demonstrated by Wright et al.^45^, plant investment in leaf traits such as leaf nutrient concentrations and photosynthetic capacity refer to the long-term adaptations to climate, especially air temperature, precipitation, and the length of growing season^34^. Our comparison of the three closely related species of Oenothera points to the following highlights which seems to be important for their biogeographical potential:

1/ *O. rubricaulis* (a species adapted to colder climate) is characterized by the highest chlorophyll content accompanied by the accumulation of anthocyanins. These features may maximize light acquisition under low light and also protect against photoinhibiton.

2/ *O. biennis* (a species with the most extended range of occurrence in Europe) possesses a lower chlorophyll content, in comparison to *O. rubricaulis*, and the highest resistance to photoinhibition under high intensity of blue light. The latter might indicate the highest plasticity of photosynthetic apparatus.

3/ *O. suaveolens* (a species adapted to warmer climate) has the highest leaf transmittance associated with the lowest chlorophyll content. These features together with the hyponasty of the leaves and the most rapid photoinhibition of Pn under blue light may point to a strategy of light avoidance, as an adaptation to the environment where light is in excess. In turn, the highest stomatal conductance might indicate the benefits of high transpiration for leaf cooling.

It should be emphasized that the morphological and physiological differentiation was detected among species having an identical nuclear and plastid-genome combination, i.e. AB-II while the previous analyses were mostly focused on species and artificial hybrids having distinct combinations. Moreover, since the history of colonization and adaptation of *O. biennis*, *O. suaveolens* and *O. rubricaulis* in Europe goes back only a few hundred years, it seems that environmental selection following the hybridization events has played an important role during the establishment of these species in Europe.

Finally, it appears that there are several contact zones between the studied species in Europe. The study of such mixed populations in nature would provide new insights into evolution of these species on the European continent in the context of hybridization. Of special interest would be also an examination of the progeny resulting from crossing experiments between particular pairs of species. Analyses of the morphology and fitness of the resulting hybrids, compared with the parental lineages, would shed a new light on evolution of this group. Moreover, they allow for the prediction of the future fates of the species, in particularly their chances of maintaining the identity in the face of the threat of hybridization.

## Materials and methods

### Plant material

Three closely related *Oenothera* species were chosen as study objects: the true (= European) *O. biennis* L., *O. rubricaulis* Kleb., and *O. suaveolens* Desf. ex Pers. All the three species (2n = 2x = 14) poses the same plastome-genome combination (AB-II), although they form different chromosome arrangements during meiosis. Three types of samples of chosen *Oenothera* species were used depending on the type of analysis, as described below.

a/ Detailed dot maps of the species’ distribution were generated on the basis of specimens stored in several herbaria (AMD, Fl, L, NCY, KRAM, KTU, P, WRSL; herbaria acronyms according to Thiers^46^), online databases^21,26^, and literature data [47,48 and literature cited therein]. The specimens were critically revised by a specialist in *Oenothera* (M. Woźniak-Chodacka). The use of plant parts in the study complies with international, national, and/or institutional guidelines. Dot maps of the distribution were generated using Simplemappr (www.simplemappr.net).

b/ Morphometric research was carried out on dried specimens mostly from the collections of Krzysztof Rostański’s and Monika Woźniak-Chodacka stored in KTU, KRAM and WRSL, in order to avoid taxonomic inconsistency in the accepted species concept within the genus. The specimens were collected from several European countries: *O. biennis* from Germany, Switzerland, Russia, Hungary, Belarus, Czech Republic, United Kingdom, Poland, Estonia, Sweden; *O. rubricaulis* from Lithuania, Latvia, Estonia, Germany, Poland, Belarus, Russia, Ukraine, Czech Republic, Austria, Belgium; and *O. suaveolens* from Italy, Poland, Hungary, Portugal, Germany, Slovakia, Czech Republic, and France. Among them, 238 most complete and undamaged specimens were chosen for further analyses. The list of deposition numbers (voucher IDs) of specimens used in this study is presented in supplementary Table S2. The list of examined characteristics consisted of 34 characters, including both quantitative and qualitative traits as well as ratio values (Table 1). A detailed description of methodology for principal component analysis (PCA), discriminant analysis (DA) and canonical discriminant analysis (CDA) are given in supplementary text.

c/ Photosynthetic parameters were evaluated on plants cultivated from seeds collected from the overlapping part of the distribution ranges of the three species in Central Europe (supplementary Table S3). This approach enable to visualize the genetically fixed traits. Ten plants per species were cultivated. Germination was conducted following the methodology established by Stephan Greiner, as described by Greiner and Kohl^49^. Post-germination, the plants were grown under controlled environmental conditions, receiving an irradiance of 200 µmol PPFD m^− 2^ s^− 1^ of red and blue light. The temperature was maintained within a range of 20 to 22 °C, and a long-day photoperiod was implemented, with 16 h of light and 8 h of darkness. Physiological research was conducted at the early rosette stage, which occurs approximately 6 to 8 weeks after germination (Fig. 1a, top panel).

### Chlorophyll content

Chlorophyll content was estimated non-invasively by SPAD (the *s*oil *p*lant *a*nalysis *d*evelopment) method. SPAD values were determined with a leaf chlorophyll meter SPAD 502 (Konica Minolta Sensing Europe B.V., Warrington, UK). The device measured the difference between the absorption of light by leaf chlorophyll at a wavelength of 650 nm and the light absorbed by the other elements of the structure at 940 nm.

### Leaf optical properties (LOPs)

LOPs were determined on dark-adapted leaves according to Grzesiak et al. 2010^50^. Reflectance (R) and transmittance (T) of leaf blade were measured using a spectroradiometer GL Spectis 5.0 Touch (GL Optic Lichtmesstechnik GmbH, Weilheim/Teck, Germany) in the 400–700 nm range, attached via an optical fibre to the externally-integrated LI-1800–12 S sphere (LI-COR Inc., Lincoln, USA) with quartz tungsten halogen lamp operating at a colour temperature of 3150 K, calibrated with a standard magnesium oxide dish as the 100% reflectance. Absorptance (A) was estimated using the following formula: A = 100 – (R + T).

### Gas exchange

The photosynthetic parameters were measured with a Li-6400XT Portable Photosynthesis System (LI-COR Inc., Lincoln, USA) with 2 × 3 cm transparent chamber (6400-08), illuminated with LED Light Source SL 3500-C, which enables red and blue actinic irradiation separately^51^, and equipped with automatic light controller (Photon Systems Instruments, Brno, Czech Republic). Measurements were performed at a constant temperature of the measurement block (30 °C), controlled CO_2_ supply (400 µmol mol^− 1^) and relative humidity (35–40%). After leaf acclimation to the cuvette environment, the photosynthetic light response curves were estimated. The light response curve measurements were conducted with a descending of red light levels (1300, 1000, 600, 200, 100, 0 µmol PPFD m^− 2^ s^− 1^) and with an ascending blue light levels (0, 50, 100, 200, 400, 600, 1000 µmol PPFD m-2 s-1). At each level, leaf gas exchange was monitored to ensure reaching steady-state plateau before data-logging. The values of net photosynthetic rate (Pn), stomatal conductance (*g*s) were recorded.

## Electronic supplementary material

Below is the link to the electronic supplementary material.

Supplementary Material 1

Supplementary Material 2

Supplementary Material 3

Supplementary Material 4

Supplementary Material 5

Supplementary Material 6

Supplementary Material 7

Supplementary Material 8

## Data Availability

The datasets generated during and/or analyzed during the current study are available from the corresponding author on responsible request.
