# Supplementary material for: Environmental selection underlies distinct distribution patterns of closely related European evening primroses
Source: Sci Rep. 2025 Feb 5;15:4436. doi: 10.1038/s41598-025-88888-3 (PMC11799430; doi:10.1038/s41598-025-88888-3)

Woźniak-Chodacka, M., Kocurek M., Pilarska, M. & Niewiadomska, E. Environmental selection underlies distinct distribution patterns of closely related European evening primroses.

Supplementary information

Figure S1. The results of principal component analysis (PCA) on all quantitative data: a - scatterplot, b - factor loadings. Each point represents a single specimen. For characters abbreviations see Table 1.

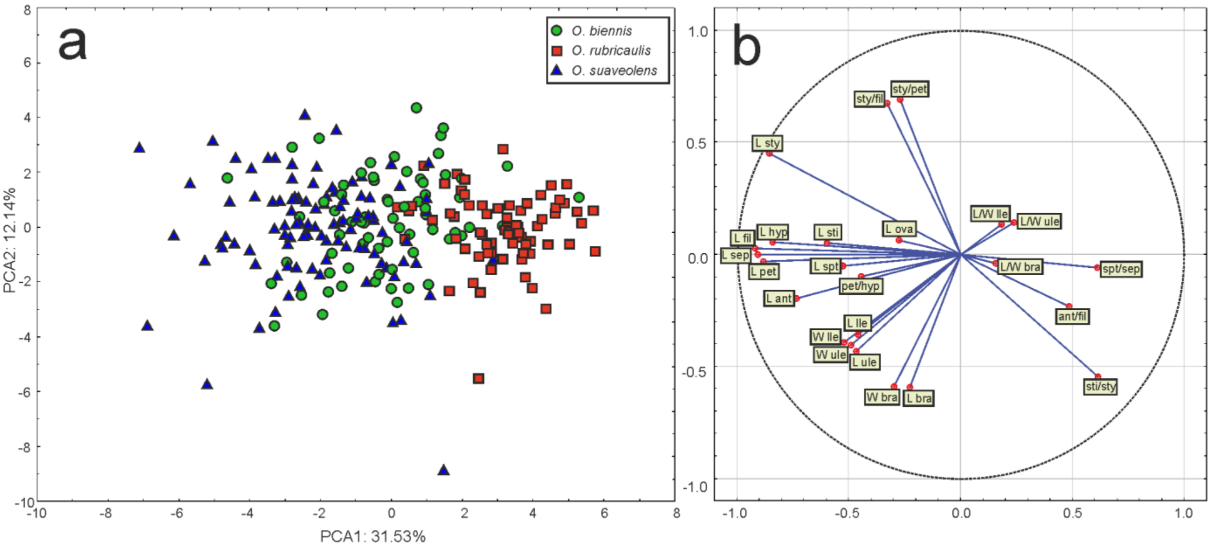

Supplement: Supplementary file 1 — Supplementary Material 1 [file 41598_2025_88888_MOESM1_ESM.pdf]
