# Supplementary material for: Environmental selection underlies distinct distribution patterns of closely related European evening primroses
Source: Sci Rep. 2025 Feb 5;15:4436. doi: 10.1038/s41598-025-88888-3 (PMC11799430; doi:10.1038/s41598-025-88888-3)

Woźniak-Chodacka, M., Kocurek M., Pilarska, M. & Niewiadomska, E. Environmental selection underlies distinct distribution patterns of closely related European evening primroses.

Supplementary information

Figure S2. The density of stomata in the three species. Letters above bars indicate statistically significant differences according to one-way ANOVA and Duncan *post hoc* test ($P < 0.05$, mean value ± SE). For estimation of stomatal density, we used 5 plants per species, one leaf per individual plant and 4 microscope fields per leaf. The abaxial epidermis, on which stomatal were more abundant, was peeled off and observations were made using the Eclipse E400 microscope (Nikon USA, Melville, NY). The stomatal density was calculated as the stomatal number per mm^2. Statistical significance was determined by analysis of variance (ANOVA), followed by Duncan *post hoc* test ($P<0.05$) using SigmaPlot 12 (Systat Software, Inc, USA).

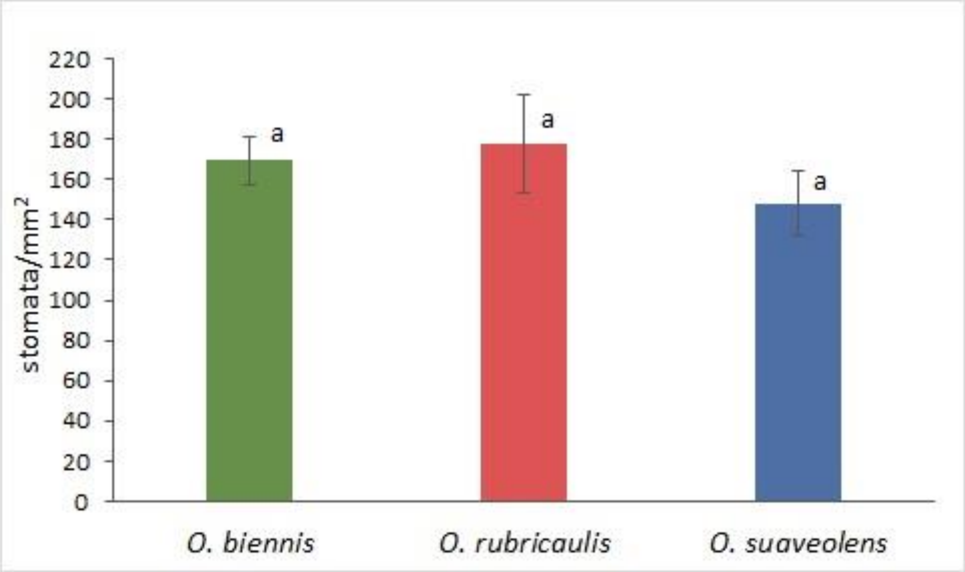

Supplement: Supplementary file 2 — Supplementary Material 2 [file 41598_2025_88888_MOESM2_ESM.pdf]
