# Supplementary material for: Environmental selection underlies distinct distribution patterns of closely related European evening primroses
Source: Sci Rep. 2025 Feb 5;15:4436. doi: 10.1038/s41598-025-88888-3 (PMC11799430; doi:10.1038/s41598-025-88888-3)

Woźniak-Chodacka, M., Kocurek M., Pilarska, M. & Niewiadomska, E. Environmental selection underlies distinct distribution patterns of closely related European evening primroses.

Supplementary information

Figure S3. A scatter diagram showing the intraspecific diversity of the three species based on results of canonical discriminant analysis (CDA) of all quantitative data. The epithets assigned to the species names refer to specimens collected from Northern, Central and Southern Europe. Each point represents one specimen.

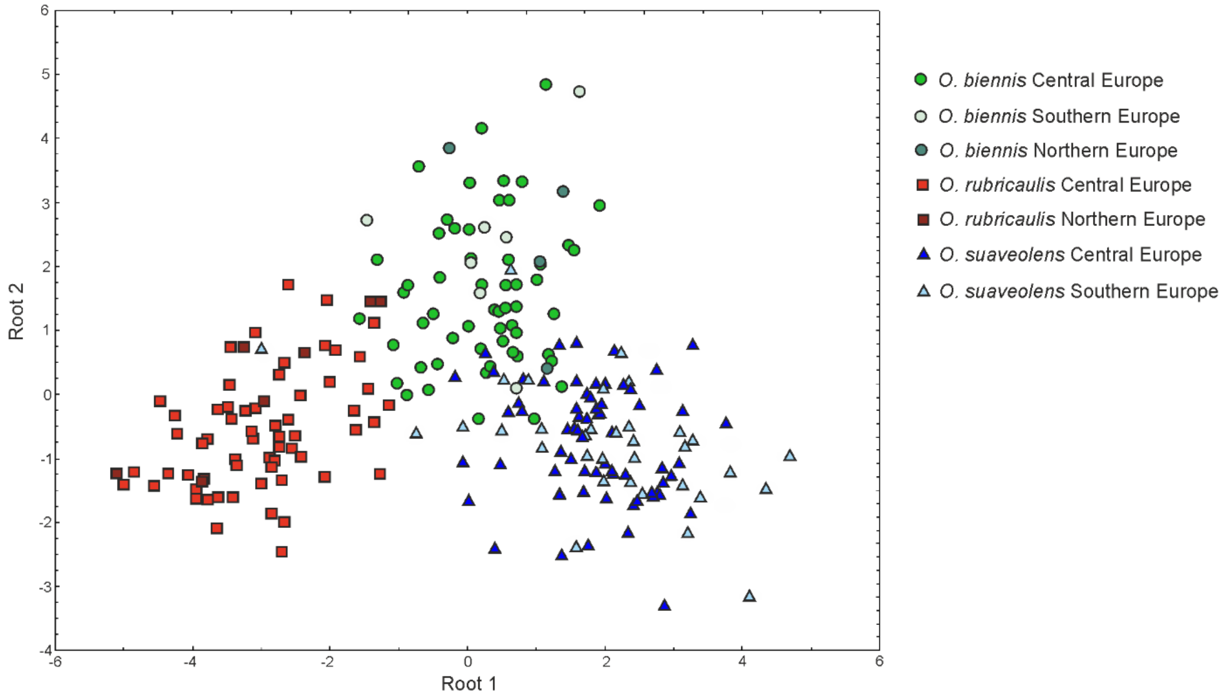

Supplement: Supplementary file 3 — Supplementary Material 3 [file 41598_2025_88888_MOESM3_ESM.pdf]
