# Supplementary material for: Environmental selection underlies distinct distribution patterns of closely related European evening primroses
Source: Sci Rep. 2025 Feb 5;15:4436. doi: 10.1038/s41598-025-88888-3 (PMC11799430; doi:10.1038/s41598-025-88888-3)

Woźniak-Chodacka, M., Kocurek M., Pilarska, M. & Niewiadomska, E. Environmental selection underlies distinct distribution patterns of closely related European evening primroses.

Supplementary information

Figure S4. A diagram showing the intraspecific diversity of the three species (OB = *O. biennis;* OR = *O. rubricaulis*; OS = *O. suaveolens*) based on the frequency of the defined states ("0" and "1") of the studied qualitative features. The capital letters assigned to the species names (N, C, S) refer to specimens collected from Northern, Central and Southern Europe, respectively. The state "0" refers to green, while "1" refers to red colour of particular organs (inflorescence axis, top of the inflorescence axis, nerves, papillae on the stem, inflorescence axis and ovaries). It also refers to the predominance of glandular ("0") vs. strigillose ("1") hairs on the selected plant structures (inflorescence axis, ovaries, hypanthia, and flower buds).

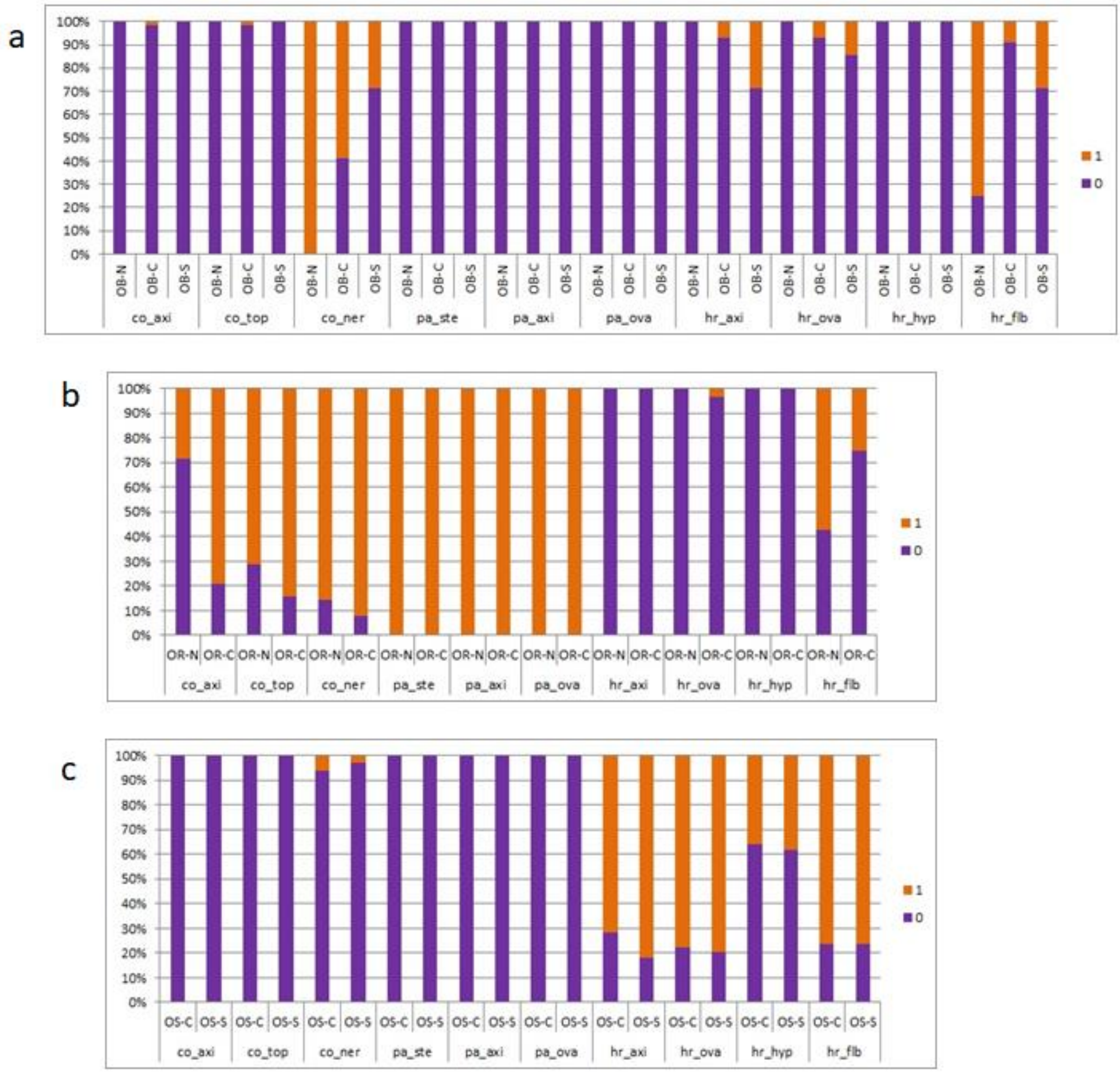

Supplement: Supplementary file 4 — Supplementary Material 4 [file 41598_2025_88888_MOESM4_ESM.pdf]
