# Supplementary material for: Environmental selection underlies distinct distribution patterns of closely related European evening primroses
Source: Sci Rep. 2025 Feb 5;15:4436. doi: 10.1038/s41598-025-88888-3 (PMC11799430; doi:10.1038/s41598-025-88888-3)
Supplement: Supplementary file 5 — Supplementary Material 5 [file 41598_2025_88888_MOESM5_ESM.pdf]

Woźniak-Chodacka, M., Kocurek M., Pilarska, M. & Niewiadomska, E. Environmental selection underlies distinct distribution patterns of closely related European evening primroses.

Supplementary information

Table S1. The results of discriminant analysis (DA) and canonical discriminant analysis (CDA). Values for which the discriminant functions are most weighted are given in bold.

Feature	DA			CDA	
	Wilks' *lambda*	Partial Wilks' *lambda*	p	Root1	Root2
LLL	0.10	0.93	**0.0004**	0.11	**-1.48**
LLW	0.10	0.92	**0.0001**	-0.07	**1.95**
ULL	0.09	1.00	0.8558	-0.12	0.00
ULW	0.09	0.99	0.4645	0.17	0.34
BL	0.10	0.90	**0.0000**	-0.32	**-1.48**
BW	0.10	0.93	**0.0004**	0.28	**1.28**
HL	0.10	0.87	**0.0000**	**0.87**	-0.23
PL	0.10	0.94	**0.0008**	**1.04**	-0.20
FL	0.09	0.97	**0.0450**	**-0.91**	**-0.75**
AL	0.09	0.97	**0.0295**	**0.68**	0.41
SLL	0.09	0.98	0.1707	**-0.77**	0.00
SGL	0.09	0.99	0.3083	-0.15	0.08
OL	0.09	0.95	0.0037	0.12	0.34
SL	0.10	0.90	**0.0000**	**-0.81**	**-1.07**
STL	0.10	0.92	**0.0002**	**0.61**	**1.27**
LLL/LLW	0.10	0.93	**0.0006**	0.00	**1.20**
ULL/ULW	0.09	0.99	0.3429	0.08	0.32
BL/BW	0.09	0.95	**0.0041**	0.11	0.52
AL/FL	0.09	0.98	0.1273	-0.44	**-0.61**
SGL/SLL	0.09	1.00	0.5880	-0.09	-0.14
SLL/FL	0.09	0.99	0.5757	-0.49	-0.28
SLL/PL	0.09	0.97	0.0247	**1.16**	0.36
PL/HL	0.09	0.97	0.0595	0.38	-0.36
STL/SL	0.11	0.84	**0.0000**	**-0.84**	**-1.55**
Eigenvalue		4.27	1.14		
Cumulative proportion [%]		0.79	1.00		
