# Supplementary material for: Environmental selection underlies distinct distribution patterns of closely related European evening primroses
Source: Sci Rep. 2025 Feb 5;15:4436. doi: 10.1038/s41598-025-88888-3 (PMC11799430; doi:10.1038/s41598-025-88888-3)
Supplement: Supplementary file 6 — Supplementary Material 6 [file 41598_2025_88888_MOESM6_ESM.pdf]

Woźniak-Chodacka, M., Kocurek M., Pilarska, M. & Niewiadomska, E. Environmental selection underlies distinct distribution patterns of closely related European evening primroses.

Supplementary information

Table S2. A table presenting the details on plant materials used for morphometric analyses.

No	Species	Herbarium	Voucher ID	Location	Latitude	Longitude	Date	Collector
1	*O. biennis*	KTU	100492	Germany, Biedenkopf	50.916	8.533	16.08.1990	W. Schnedler
2	*O. biennis*	KTU	100494	Switzerland, Bern	46.953	7.454	4.08.1999	K. Rostański
3	*O. biennis*	KTU	100503	Russia, Nikiel	44.177	40.155	28.06.1991	K. Rostański
4	*O. biennis*	KTU	100505	Russia, Myakop	44.806	40.113	28.06.1991	K. Rostański
5	*O. biennis*	KTU	100504	Russia, Myakop	44.806	40.113	28.06.1991	K. Rostański
6	*O. biennis*	KTU	100491	Germany, Grossostheim	49.916	9.0833	19.09.1990	W. Schnedler
7	*O. biennis*	KTU	52235	Hungary, Budapest	47.471	19.050	8.08.1964	K. Rostański
8	*O. biennis*	KTU	100749	Belarus, Ostrovok	53.151	27.254	24.07.2002	K. Rostański
9	*O. biennis*	KTU	100756	Czech Republic, Třinec	49.677	18.672	15.07.1992	K. Rostański & V. Jehlik
10	*O. biennis*	KTU	100751	Russia, Koltsovo	54.499	36.404	3.09.1971	W. Makarow & G. Proskurjakowa
11	*O. biennis*	KTU	100755	Russia, Veshenskaya	49.616	41.716	3.07.1991	K. Rostański
12	*O. biennis*	KTU	100747	England, Longmoor	51.083	-0.857	15.07.1978	A. Brevis
13	*O. biennis*	KTU	100753	Russia, Veshenskaya	49.616	41.716	3.07.1991	K. Rostański
14	*O. biennis*	KTU	100556	Poland, Hureczko	49.785	22.834	20.07.2000	K. Rostański
15	*O. biennis*	KTU	100568	Poland, Białowieża	52.702	23.858	7.08.1997	K. Rostański
16	*O. biennis*	KTU	100412	Poland, Wolbrom	50.385	19.758	20.07.1978	K. Rostański
17	*O. biennis*	KTU	100419	Poland, Łozice	52.667	23.574	17.08.1987	L. Bernacki
18	*O. biennis*	KTU	131519	Poland, Borne Sulinowo	53.583	16.541	29.06.2009	A. Rostański
19	*O. biennis*	KTU	130008	Germany, Mecklenburg	53.834	11.466	23.07.2008	H. Henker & H. Kiesewetter

20	*O. biennis*	KTU	100428	Poland, Biłgoraj	50.540	22.721	2.09.1988	K. Rostański
21	*O. biennis*	KTU	52286	Poland, Toruń	53.010	18.604	11.07.1994	K. Rostański
22	*O. biennis*	KTU	51412	Switzerland, Zürich	47.366	8.552	12.07.1997	E. Landolt
23	*O. biennis*	KTU	100472	Poland, Kielce	50.819	20.555	30.07.1985	K. Rostański
24	*O. biennis*	KTU	100075	Russia, Rostov	47.233	39.722	24.09.1987	K. Rostański
25	*O. biennis*	KTU	100484	Poland, Korytków Duży	50.623	22.668	2.09.1988	K. Rostański
26	*O. biennis*	KTU	100498	Estonia, Tallinn	59.433	24.754	11.07.1999	K. Rostański
27	*O. biennis*	KTU	100499	Estonia, Tallinn	59.433	24.754	11.07.1999	K. Rostański
28	*O. biennis*	KTU	131550	Germany, Kehl	48.566	7.816	23.08.1980	K. Rostański
29	*O. biennis*	KTU	100746	England, Longmoor	51.083	-0.857	15.07.1978	A. Brevis
30	*O. biennis*	KTU	100732	Germany, Delbrück	51.766	8.566	9.09.1995	K. Rostański
31	*O. biennis*	KTU	131555	Russia, Adler	43.447	39.914	19.06.1990	K. Rostański
32	*O. biennis*	KTU	130007	Germany, Mecklenburg	53.834	11.466	23.07.2008	H. Henker & H. Kiesewetter
33	*O. biennis*	KRAM	653776	Poland, Kraków	50.059	20.007	26.07.2010	M. Woźniak-Chodacka
34	*O. biennis*	KRAM	653777	Poland, Kraków	50.059	20.007	26.07.2010	M. Woźniak-Chodacka
35	*O. biennis*	KRAM	653778	Poland, Jaworzno	50.203	19.283	26.07.2010	M. Woźniak-Chodacka
36	*O. biennis*	KTU	52265	Germany, Essen	51.450	7.013	20.07.1992	T. Kalveram
37	*O. biennis*	KRAM	653779	Poland, Kraków	50.059	20.007	26.07.2010	M. Woźniak-Chodacka
38	*O. biennis*	KTU	130006	Germany, Mecklenburg	53.834	11.466	16.07.2008	H. Henker & H. Kiesewetter
39	*O. biennis*	KRAM	653780	Poland, Kraków	50.059	20.007	26.07.2010	M. Woźniak-Chodacka
40	*O. biennis*	KTU	100745	England, Longmoor	51.083	-0.857	15.07.1978	A. Brevis
41	*O. biennis*	KRAM	653781	Poland, Jaworzno	50.203	19.283	19.07.2019	M. Woźniak-Chodacka
42	*O. biennis*	KTU	100744	England, Longmoor	51.083	-0.857	15.07.1978	A. Brevis
43	*O. biennis*	KTU	100435	Poland, Siemianowice Śląskie	50.307	19.025	10.07.1997	K. Bzdęga
44	*O. biennis*	WRSL	s.n.	Sweden, Simrishamn	55.552	14.344	16.06.1981	E. Kozioł
45	*O. biennis*	KRAM	653782	Germany, Golm	52.414	12.970	12.07.2019	M. Woźniak-Chodacka
46	*O. biennis*	KRAM	653783	Germany, Golm	52.414	12.970	12.07.2019	M. Woźniak-Chodacka
47	*O. biennis*	KRAM	653784	Poland, Jaworzno	50.203	19.283	19.07.2019	M. Woźniak-Chodacka
48	*O. biennis*	KRAM	653785	Poland, Tychy	50.131	19.010	19.07.2019	M. Woźniak-Chodacka
49	*O. biennis*	KRAM	653786	Poland, Żabno	50.136	20.887	23.07.2019	M. Woźniak-Chodacka
50	*O. biennis*	KRAM	653787	Poland, Podlesie Dębowe	50.158	20.871	23.07.2019	M. Woźniak-Chodacka
51	*O. biennis*	KRAM	653788	Poland, Kraków	50.059	20.007	24.07.2019	M. Woźniak-Chodacka

52	*O. biennis*	KRAM	653789	Poland, Kraków	50.059	20.007	24.07.2019	M. Woźniak-Chodacka
53	*O. biennis*	KRAM	653790	Poland, Kraków	50.059	20.007	24.07.2019	M. Woźniak-Chodacka
54	*O. biennis*	KRAM	653791	Poland, Nowy Sącz	49.587	20.702	27.07.2019	M. Woźniak-Chodacka
55	*O. biennis*	KRAM	653792	Poland, Wierzchosławice	50.034	20.816	7.09.2019	M. Woźniak-Chodacka
56	*O. biennis*	KRAM	653793	Germany, München	48.120	11.540	8.07.2019	M. Woźniak-Chodacka
57	*O. biennis*	KRAM	653794	Germany, München	48.120	11.540	8.07.2019	M. Woźniak-Chodacka
58	*O. biennis*	KRAM	653795	Russia, Volgograd	48.732	44.590	9.07.2019	M. Woźniak-Chodacka
59	*O. biennis*	KRAM	653796	Russia, Volgograd	48.732	44.590	9.07.2019	M. Woźniak-Chodacka
60	*O. biennis*	KRAM	653797	France, Rumersheim	48.689	7.637	10.07.2019	M. Woźniak-Chodacka
61	*O. biennis*	KRAM	653798	Poland, Żabno	50.136	20.887	23.07.2019	M. Woźniak-Chodacka
62	*O. biennis*	KRAM	653799	Germany, Friedrichshagen	52.463	13.642	8.07.2019	M. Woźniak-Chodacka
63	*O. biennis*	KRAM	653800	Germany, Friedrichshagen	52.463	13.642	8.07.2019	M. Woźniak-Chodacka
64	*O. biennis*	KRAM	653801	Poland, Bukowno	50.263	19.426	16.07.2019	M. Woźniak-Chodacka
65	*O. biennis*	KRAM	653802	Poland, Bukowno	50.263	19.426	16.07.2019	M. Woźniak-Chodacka
66	*O. biennis*	KRAM	653803	Poland, Podlesie Dębowe	50.158	20.871	23.07.2019	M. Woźniak-Chodacka
67	*O. biennis*	KRAM	653804	Germany, Luckenwalde	52.098	13.171	8.07.2019	M. Woźniak-Chodacka
68	*O. suaveolens*	KTU	103576	Germany, Seeheim	49.750	8.650	24.07.1990	K. Rostański
69	*O. suaveolens*	KTU	103577	Germany, Seeheim	49.750	8.650	24.07.1990	K. Rostański & W. Schnedler
70	*O. suaveolens*	KTU	51695	Poland,Brzózka Krośnieńska	51.998	15.029	21.07.1961	K. Rostański
71	*O. suaveolens*	KTU	51702	Poland, Wrocław	51.103	17.029	21.08.1963	K. Rostański
72	*O. suaveolens*	KTU	51704	Poland, Wrocław	51.103	17.029	8.08.1961	K. Rostański
73	*O. suaveolens*	KTU	51704	Poland, Wrocław	51.103	17.029	8.08.1961	K. Rostański
74	*O. suaveolens*	KTU	51704	Poland, Wrocław	51.103	17.029	8.08.1961	K. Rostański
75	*O. suaveolens*	KTU	51705	Hungary, Győr	47.684	17.634	21.08.1964	K. Rostański
76	*O. suaveolens*	KTU	51706	Hungary, Szolnok	47.174	20.176	13.08.1964	K. Rostański
77	*O. suaveolens*	KTU	51707	Hungary, Szajol	47.183	20.302	28.07.1959	K. Rostański
78	*O. suaveolens*	KTU	52405	Portugal, Faial	38.577	-28.703	19.06.1999	H. Schaefer
79	*O. suaveolens*	KTU	52718	Poland, Puszczykowo	52.272	16.858	20.08.1979	K. Rostański
80	*O. suaveolens*	KTU	60029	Germany, Berlin	52.518	13.408	4.07.1997	B. Tokarska-Guzik
81	*O. suaveolens*	KTU	103567	Italy, Vercelli	45.316	8.416	22.08.1981	A. Soldano
82	*O. suaveolens*	KTU	103568	Italy, Vercelli	45.316	8.416	9.09.1983	A. Soldano
83	*O. suaveolens*	KTU	103569	Italy, Albano Vercellese	45.433	8.383	26.08.2000	K. Rostański

84	*O. suaveolens*	KTU	103570	Italy, Albano Vercellese	45.433	8.383	26.08.2000	K. Rostański
85	*O. suaveolens*	KTU	103571	Italy, Albano Vercellese	45.433	8.383	26.08.2000	K. Rostański
86	*O. suaveolens*	KTU	103572	Italy, Albano Vercellese	45.433	8.383	26.08.2000	K. Rostański
87	*O. suaveolens*	KTU	103575	Germany, Seeheim	49.752	8.655	24.07.1990	K. Rostański
88	*O. suaveolens*	KTU	103576	Germany, Seeheim	49.752	8.655	24.07.1990	K. Rostański
89	*O. suaveolens*	KTU	103577	Germany, Seeheim	49.752	8.655	24.07.1990	K. Rostański& W. Schnedler
90	*O. suaveolens*	KTU	103578	Germany, Bickenbach	49.766	8.616	24.07.1990	K. Rostański& W. Schnedler
91	*O. suaveolens*	KTU	103581	Germany, Kaefertal	49.488	8.469	16.07.1992	W. Schnedler
92	*O. suaveolens*	KTU	103579	Germany, Jaegersburg	49.369	7.323	13.08.1991	W. Schnedler
93	*O. suaveolens*	KTU	103582	Germany, Wilgartswiesen	49.209	7.879	22.07.1994	W. Lang
94	*O. suaveolens*	KTU	103583	Germany, Weisenheim	49.511	8.150	17.08.1994	W. Lang
95	*O. suaveolens*	KTU	103584	Germany, Weisenheim	49.511	8.150	17.08.1994	W. Lang
96	*O. suaveolens*	KTU	103585	Germany, Germersheim	49.216	8.366	27.08.1994	W. Lang
97	*O. suaveolens*	KTU	103586	Germany, Germersheim	49.216	8.366	27.08.1994	W. Lang
98	*O. suaveolens*	KTU	103587	Germany, Altrip	49.432	8.503	29.09.1994	W. Lang
99	*O. suaveolens*	KTU	103588	France, Strasbourg	48.583	7.733	20.08.1994	W. Lang
100	*O. suaveolens*	KTU	103589	France, Wissembourg	49.033	7.950	22.06.2000	W. Lang
101	*O. suaveolens*	KTU	103590	Slovakia, Sekule	48.599	17.003	24.07.1973	K. Rostański
102	*O. suaveolens*	KTU	103591	Czech Republic, Třinec	49.677	18.672	15.07.1992	K. Rostański & V. Jehlik
103	*O. suaveolens*	KTU	103592	Czech Republic, Třinec	49.677	18.672	15.07.1992	K. Rostański & V. Jehlik
104	*O. suaveolens*	KTU	103593	Czech Republic, Třinec	49.677	18.672	15.07.1992	K. Rostański & V. Jehlik
105	*O. suaveolens*	KTU	103594	Czech Republic, Třinec	49.677	18.672	15.07.1992	K. Rostański & V. Jehlik
106	*O. suaveolens*	KTU	103595	Hungary, Szajol	47.183	20.302	28.07.1959	K. Rostański
107	*O. suaveolens*	KTU	103601	Hungary, Dunaharaszti	47.353	19.094	21.08.1964	K. Rostański
108	*O. suaveolens*	KTU	103601	Hungary, Dunaharaszti	47.353	19.094	21.08.1964	K. Rostański
109	*O. suaveolens*	KTU	103602	Hungary, Győr	47.684	17.634	21.08.1964	K. Rostański
110	*O. suaveolens*	KTU	103603	Poland, Wrocław	51.103	17.029	8.08.1961	K. Rostański
111	*O. suaveolens*	KTU	103610	Hungary, Szentpeterszeg	42.246	21.617	8.07.1968	K. Rostański
112	*O. suaveolens*	KTU	103612	Hungary, Hencida	47.254	21.708	2.07.1968	K. Rostański
113	*O. suaveolens*	KTU	103619	Poland, Mosina	52.243	16.850	4.07.2001	K. Rostański
114	*O. suaveolens*	KTU	103621	Poland, Lublin	51.247	22.560	7.09.1990	K. Rostański
115	*O. suaveolens*	KTU	103623	Poland, Lublin	51.247	22.560	7.09.1990	K. Rostański

116	*O. suaveolens*	KTU	103626	Italy, Rosolina Mare	45.134	12.323	14.08.1996	J. Mol
117	*O. suaveolens*	KTU	103628	Italy, Rosolina Mare	45.134	12.323	14.08.1996	J. Mol
118	*O. suaveolens*	KTU	103629	Italy, Rosolina Mare	45.134	12.323	14.08.1996	J. Mol
119	*O. suaveolens*	KTU	103643	Poland, Zabrze	50.342	18.813	4.07.1996	E. Holak
120	*O. suaveolens*	KTU	103651	Poland, Zabrze	50.342	18.813	4.07.1996	E. Holak
121	*O. suaveolens*	KTU	130032	Germany, Mecklenburg	53.834	11.466	16.07.2008	H. Henker & H. Kiesewetter
122	*O. suaveolens*	KTU	130033	Germany, Mecklenburg	53.834	11.466	8.08.2007	H. Henker & H. Kiesewetter
123	*O. suaveolens*	KTU	130034	Germany, Mecklenburg	53.834	11.466	5.08.2008	H. Henker & H. Kiesewetter
124	*O. suaveolens*	KTU	130035	Germany, Mecklenburg	53.834	11.466	7.08.2007	H. Henker & H. Kiesewetter
125	*O. suaveolens*	KTU	130036	Germany, Mecklenburg	53.834	11.466	8.07.2007	H. Henker & H. Kiesewetter
126	*O. suaveolens*	KTU	130880	Germany, Kehl	48.566	7.816	23.08.1980	K. Rostański
127	*O. suaveolens*	KTU	130880	Germany, Kehl	48.566	7.816	23.08.1980	K. Rostański
128	*O. suaveolens*	KTU	130882	Germany, Marlen am Rhein	48.520	7.827	23.08.1980	K. Rostański
129	*O. suaveolens*	KTU	130883	Germany, Marlen am Rhein	48.520	7.827	23.08.1980	K. Rostański
130	*O. suaveolens*	KTU	130884	Germany, Ottenheim	48.386	7.757	24.08.1980	K. Rostański
131	*O. suaveolens*	KTU	130888	Germany, Wünsdorf	52.163	13.474	7.07.1979	K. Rostański
132	*O. suaveolens*	KTU	130892	Poland, Puszczykówko	52.275	16.853	21.08.1979	K. Rostański
133	*O. suaveolens*	KTU	130893	Poland, Puszczykówko	52.275	16.853	21.08.1979	K. Rostański
134	*O. suaveolens*	KTU	130894	Poland, Puszczykówko	52.275	16.853	21.08.1979	K. Rostański
135	*O. suaveolens*	KTU	130895	Italy, Rosolina Mare	45.134	12.323	10.08.1996	J. Mol
136	*O. suaveolens*	KTU	130897	Italy, Rosolina Mare	45.134	12.323	31.08.1996	J. Mol
137	*O. suaveolens*	KTU	130899	Italy, Bibione	45.766	12.854	29.07.1996	B. Tokarska-Guzik
138	*O. suaveolens*	KTU	130903	Germany, Annweiler	49.203	7.966	7.07.1993	W. Lang
139	*O. suaveolens*	KTU	130907	Poland, Lublin	51.247	22.560	7.09.1990	K. Rostański
140	*O. suaveolens*	KTU	130908	Italy, Rosolina Mare	45.134	12.323	28.08.1995	K. Rostański
141	*O. suaveolens*	KTU	130909	Italy, Rosolina Mare	45.134	12.323	31.08.1995	K. Rostański
142	*O. suaveolens*	KTU	130910	Italy, Rosolina Mare	45.134	12.323	1.09.1995	K. Rostański
143	*O. suaveolens*	KTU	130912	Italy, Rosolina Mare	45.134	12.323	2.09.1995	K. Rostański
144	*O. suaveolens*	KTU	130913	Italy, Rosolina Mare	45.134	12.323	2.09.1995	K. Rostański
145	*O. suaveolens*	KTU	130914	France, Port Galland	45.766	4.833	3.08.1988	K. Rostański
146	*O. suaveolens*	KTU	130915	France, Saint-Père-sur-Loire	47.770	2.369	31.07.1988	K. Rostański
147	*O. suaveolens*	KTU	130916	France, Saint-Père-sur-Loire	47.770	2.369	31.07.1988	K. Rostański

148	*O. suaveolens*	KTU	130917	France, Saint-Père-sur-Loire	47.770	2.369	31.07.1988	K. Rostański
149	*O. suaveolens*	KTU	130924	Germany, Neurath	51.039	6.615	23.07.1976	K. Rostański & W. Dietrich
150	*O. suaveolens*	KTU	131415	Germany, Wünsdorf	52.163	13.47	27.06.1967	K. Rostański
151	*O. suaveolens*	KTU	131946	Poland, Katowice	50.266	19.029	19.06.2011	Ł. Fołcik
152	*O. suaveolens*	FI	50633	Italy, Presso	45.708	10.119	1890	--
153	*O. suaveolens*	FI	50625	Italy, Viareggio	43.876	10.241	10.1928	M. Tani
154	*O. suaveolens*	FI	50636	France, Bayonne	43.493	-1.478	10.1887	A. Autheman
155	*O. suaveolens*	FI	50635	Italy, Roseto	42.675	14.014	06.1949	G. Zodda
156	*O. suaveolens*	FI	50623	Italy, Donnas	45.598	7.768	22.09.2009	A. Soldano & D. Bouvet
157	*O. suaveolens*	KRAM	653805	France, Seine-et-Marne	48.713	3.044	9.07.2019	M. Woźniak-Chodacka
158	*O. suaveolens*	KRAM	653806	France, Seine-et-Marne	48.713	3.044	9.07.2019	M. Woźniak-Chodacka
159	*O. suaveolens*	KRAM	653807	Italy, Grado	45.679	13.383	8.07.2019	M. Woźniak-Chodacka
160	*O. suaveolens*	KRAM	653808	Italy, Grado	45.679	13.383	8.07.2019	M. Woźniak-Chodacka
161	*O. suaveolens*	KRAM	653809	Hungary, Pécs	46.089	18.250	10.07.2019	M. Woźniak-Chodacka
162	*O. suaveolens*	KRAM	653810	Hungary, Pécs	46.089	18.250	10.07.2019	M. Woźniak-Chodacka
163	*O. suaveolens*	KRAM	653811	Germany, Friedrichshagen	52.460	13.632	11.07.2019	M. Woźniak-Chodacka
164	*O. suaveolens*	KRAM	653812	Germany, Friedrichshagen	52.460	13.632	11.07.2019	M. Woźniak-Chodacka
165	*O. suaveolens*	KRAM	653813	Germany, Friedrichshagen	52.460	13.632	11.07.2019	M. Woźniak-Chodacka
166	*O. suaveolens*	KRAM	653814	Italy, Vercelli	45.321	8.418	8.07.2019	M. Woźniak-Chodacka
167	*O. suaveolens*	KRAM	653815	Italy, Vercelli	45.321	8.418	8.07.2019	M. Woźniak-Chodacka
168	*O. suaveolens*	KRAM	653816	Italy, Vercelli	45.321	8.418	8.07.2019	M. Woźniak-Chodacka
169	*O. rubricaulis*	KTU	103354	Latvia, Ryga	56.966	24.133	6.07.1988	L. Bernacki
170	*O. rubricaulis*	KTU	132014	Germany, Gross Beuchow	51.852	13.894	11.07.1972	H.-W. Otto
171	*O. rubricaulis*	KTU	52494	Lithuania, Druskininkai	54.016	23.966	20.07.2000	K. Zarzycki
172	*O. rubricaulis*	KTU	103277	Poland, Sosnowiec	50.230	19.163	24.07.1980	K. Rostański
173	*O. rubricaulis*	KTU	102820	Poland, Czerwonak	52.463	16.981	5.07.2001	K. Rostański
174	*O. rubricaulis*	KTU	51626	Poland, Wrocław	51.103	17.029	8.07.1958	K. Rostański
175	*O. rubricaulis*	KTU	51630	Poland, Wrocław	51.103	17.029	24.06.1959	K. Rostański
176	*O. rubricaulis*	KTU	102763	Poland, Wrocław	51.103	17.029	11.07.1960	K. Rostański
177	*O. rubricaulis*	KTU	55326	Poland, Zawiercie	50.491	19.424	30.07.1993	A. Piedo
178	*O. rubricaulis*	KTU	32351	Poland, Wrocław	51.103	17.029	25.06.1969	K. Rostański
179	*O. rubricaulis*	KTU	103288	Poland, Częstochowa	50.812	19.112	17.07.1997	K. Rostański

180	*O. rubricaulis*	KTU	103302	Poland, Bielsko-Biała	49.793	19.049	28.07.1998	B. Baron
181	*O. rubricaulis*	KTU	103063	Poland, Orzeszkowo	52.288	17.381	17.08.1987	L. Bernacki
182	*O. rubricaulis*	KTU	103343	Lithuania, Maksymonis	54.840	24.055	26.07.2002	K. Rostański
183	*O. rubricaulis*	KTU	103344	Lithuania, Merkinė	54.163	24.186	26.07.2002	K. Rostański
184	*O. rubricaulis*	KTU	103342	Lithuania, Druskininkai	54.016	23.966	25.07.2002	K. Rostański
185	*O. rubricaulis*	KTU	131611	Belgium, Ghent	51.053	3.720	26.10.2001	F. Verloove
186	*O. rubricaulis*	KTU	103426	Austria, Wien	48.216	16.366	1.07.1967	W. Forstner
187	*O. rubricaulis*	KTU	103347	Lithuania, Vilnius	54.683	25.283	16.07.2003	K. Rostański
188	*O. rubricaulis*	KTU	103348	Lithuania, Vilnius	54.683	25.283	16.07.2003	K. Rostański
189	*O. rubricaulis*	KTU	103349	Lithuania, Spindžius	54.562	24.701	17.07.2003	K. Rostański
190	*O. rubricaulis*	KTU	103357	Latvia, Ryga	56.966	24.133	11.07.1999	K. Rostański
191	*O. rubricaulis*	KTU	103362	Estonia, Tallinn	59.433	24.754	7.07.1999	K. Rostański
192	*O. rubricaulis*	KTU	51899	Germany, Leipzig	51.333	12.383	21.06.1967	K. Rostański & P. Gutte
193	*O. rubricaulis*	KTU	103419	Slovakia, Žilina	49.222	18.744	10.07.1974	V. Jehlik
194	*O. rubricaulis*	KTU	103164	Poland, Myszków	50.570	19.314	27.07.2001	K. Rostański
195	*O. rubricaulis*	KTU	103062	Poland, Wrocław	51.103	17.029	26.06.1959	K. Rostański
196	*O. rubricaulis*	KTU	103312	Poland, Orzesze	50.143	18.775	17.08.1980	M. Szczepka & P. Grzegorzek
197	*O. rubricaulis*	KTU	103002	Poland, Wiączyn	51.760	19.610	12.07.1985	K. Rostański
198	*O. rubricaulis*	KTU	130003	Germany, Mecklenburg	53.834	11.466	16.07.2008	H. Henker
199	*O. rubricaulis*	KTU	130002	Germany, Mecklenburg	53.834	11.466	7.07.2005	H. Henker
200	*O. rubricaulis*	KTU	103417	Czech Republic, Neratovice	50.251	14.522	7.07.1074	V. Jehlik
201	*O. rubricaulis*	KTU	103234	Poland, Mysłowice	50.225	19.133	16.07.1973	K. Rostański
202	*O. rubricaulis*	KTU	8104	Poland, Ostrowiec Świętokrzyski	50.938	21.389	13.08.1986	K. Rostański
203	*O. rubricaulis*	KTU	51657	Poland, Grabina Śląska	50.953	16.329	6.07.1962	K. Rostański
204	*O. rubricaulis*	KTU	35136	Poland, Mysłowice	50.225	19.133	14.08.1980	K. Rostański
205	*O. rubricaulis*	KTU	131638	Poland, Borne Sulinowo	53.583	16.541	2.07.2009	K. Rostański
206	*O. rubricaulis*	KTU	52065	Poland, Wrocław	51.103	17.029	14.06.1960	K. Rostański
207	*O. rubricaulis*	KTU	51875	Poland, Wrocław	51.103	17.029	26.06.1959	K. Rostański
208	*O. rubricaulis*	KTU	35140	Poland, Mysłowice	50.225	19.133	14.08.1980	K. Rostański
209	*O. rubricaulis*	KTU	103324	Poland, Łódź	51.77	19.455	16.07.1990	K. Rostański
210	*O. rubricaulis*	KTU	35137	Poland, Mysłowice	50.225	19.133	14.08.1980	K. Rostański

211	*O. rubricaulis*	KTU	62351	Poland, Piekary Śląskie	50.382	18.943	30.06.2000	D. Kapa
212	*O. rubricaulis*	KTU	8168	Poland, Ostrów Wielkopolski	51.646	17.808	14.07.1973	S. Wika
213	*O. rubricaulis*	KTU	103299	Poland, Pietrzykowice	49.703	19.178	5.08.1989	K. Rostański
214	*O. rubricaulis*	KTU	103233	Poland, Mysłowice	50.225	19.133	7.07.1981	K. Rostański
215	*O. rubricaulis*	KTU	131721	Poland, Piekary Śląskie	50.382	18.943	22.06.2010	K. Rostański
216	*O. rubricaulis*	KTU	131722	Poland, Katowice	50.229	18.944	22.06.2010	K. Rostański
217	*O. rubricaulis*	KTU	131718	Poland, Piekary Śląskie	50.382	18.943	22.06.2010	K. Rostański
218	*O. rubricaulis*	KTU	131524	Poland, Mysłowice	50.225	19.133	21.07.2009	K. Rostański
219	*O. rubricaulis*	KTU	42897	Poland, Klimczyce	52.356	22.846	29.06.1984	K. Rostański
220	*O. rubricaulis*	KTU	131375	Poland, Pawłowice Śląskie	49.963	18.717	10.07.1981	K. Rostański
221	*O. rubricaulis*	KTU	131445	Poland, Gdynia	54.519	18.542	29.07.1968	K. Rostański
222	*O. rubricaulis*	KTU	52495	Poland, Suwałki	54.101	22.928	10.07.2000	K. Zarzycki
223	*O. rubricaulis*	KTU	51876	Poland, Wrocław	51.103	17.029	5.07.1965	K. Rostański
224	*O. rubricaulis*	KTU	37704	Poland, Dąbrowa Górnicza	50.317	19.195	15.07.1983	K. Rostański
225	*O. rubricaulis*	KTU	60286	Poland, Budy	52.730	23.733	7.08.1997	B. Tokarska-Guzik
226	*O. rubricaulis*	KTU	103237	Poland, Mysłowice	50.225	19.133	30.06.1988	K. Rostański
227	*O. rubricaulis*	KTU	103231	Poland, Mysłowice	50.225	19.133	24.09.1980	K. Rostański
228	*O. rubricaulis*	KTU	103247	Poland, Szczyrk	49.717	19.032	8.07.1977	R. Ciepał
229	*O. rubricaulis*	KTU	102994	Poland, Białystok	53.138	23.143	11.08.1997	K. Rostański
230	*O. rubricaulis*	KTU	131242	Poland, Chruszczobród	50.415	19.317	17.06.1986	J. Skrzypek
231	*O. rubricaulis*	KTU	28209	Poland, Wrocław	51.103	17.029	11.07.1960	K. Rostański
232	*O. rubricaulis*	KTU	8147	Poland, Świerklaniec	50.442	18.936	12.07.1989	K. Rostański
233	*O. rubricaulis*	KRAM	653817	Poland, Toruń	52.993	18.628	12.07.2019	M. Woźniak-Chodacka
234	*O. rubricaulis*	KRAM	653818	Poland, Toruń	52.99	18.628	12.07.2019	M. Woźniak-Chodacka
235	*O. rubricaulis*	KRAM	653819	Poland, Toruń	52.993	18.628	12.07.2019	M. Woźniak-Chodacka
236	*O. rubricaulis*	KRAM	653820	Sweden, Nyköping	58.758	16.993	8.07.2019	M. Woźniak-Chodacka
237	*O. rubricaulis*	KRAM	653821	Poland, Giedlarowa	50.222	22.401	22.07.2019	B. Paszko
238	*O. rubricaulis*	KRAM	653822	Poland, Giedlarowa	50.222	22.401	22.07.2019	B. Paszko
