# Supplementary material for: Environmental selection underlies distinct distribution patterns of closely related European evening primroses
Source: Sci Rep. 2025 Feb 5;15:4436. doi: 10.1038/s41598-025-88888-3 (PMC11799430; doi:10.1038/s41598-025-88888-3)
Supplement: Supplementary file 7 — Supplementary Material 7 [file 41598_2025_88888_MOESM7_ESM.pdf]

Woźniak-Chodacka, M., Kocurek M., Pilarska, M. & Niewiadomska, E. Environmental selection underlies distinct distribution patterns of closely related European evening primroses.

Supplementary information

Table S3. A table detailing the plant materials used for cultivation and physiological analyses.

Species	Locality	Latitude	Longitude	Date	Collector
O. biennis	Poland, Kraków	50.073	20.005	19.07.2019	M. Woźniak-Chodacka
O. rubricaulis	Poland, Bałtów	51.007	21.540	19.08.2019	M. Woźniak-Chodacka
O. suaveolens	Poland, Jaworzno	50.243	19.304	25.07.2019	M. Woźniak-Chodacka
