# Supplementary material for: Environmental selection underlies distinct distribution patterns of closely related European evening primroses
Source: Sci Rep. 2025 Feb 5;15:4436. doi: 10.1038/s41598-025-88888-3 (PMC11799430; doi:10.1038/s41598-025-88888-3)
Supplement: Supplementary file 8 — Supplementary Material 8 [file 41598_2025_88888_MOESM8_ESM.pdf]

Woźniak-Chodacka, M., Kocurek M., Pilarska, M. & Niewiadomska, E. Environmental selection underlies distinct distribution patterns of closely related European evening primroses.

Supplementary information

Description of methodology and results presented on supplementary Table S1 and on supplementary Figs. S1, S3 and S4.

Qualitative features were binary coded and referred to the colour of the main nerves, inflorescence axis in the lower part and the top, the colour of papillae covering the stem, inflorescence axis and ovaries, as well as and to hairiness of particular organs, such as inflorescence axis, ovaries, hypanthia and flower buds. Quantitative traits were all measured in mm and encompassed length and width of lower and upper stem leaves and bracts, the length of hypanthia, petals, filaments, anthers, style, stigma, ovaries, sepals and sepal tips, as well as several ratio values (lower leaves length/width, upper leaves length/width, bracts length/width, anther/filament length, stigma/style length, style/filament length, style/petal length, petal/hypanthium length, sepal tip/sepal length).

Due to the fact that the studied taxa are very closely related, they share a considerable number of features, such as straight top of the inflorescence axis, appressed sepal tips and obcordate petals. Among the characters, there are some characteristics traditionally considered as the most reliable in *Oenothera* species delimitation, but had to be excluded since they do not differentiate among the studied taxa.

All data analyses were performed using the Statistica (StatSoft, Inc.). The distinguishing power of qualitative and quantitative characters was tested separately. At first, correspondence analysis (CA) on all binary coded qualitative features was carried out. This method allows to obtain a two-dimensional display of data set as well as to test which qualitative characters contribute the most in the observed differentiation. Next, principal component analysis (PCA) on the correlation matrix was conducted on all quantitative features to examine the general variation among specimens, as well as to reduce the set of variables to the strongest components. Main factors were obtained with the screen test. Characters with the highest factor loadings ($r>0,60$) were detected. Next, discriminant analysis (DA) followed by canonical discriminant analysis (CDA) on the matrix of all the quantitative characteristics were conducted. These analyses allow to determine which variables discriminate among *a priori* defined groups. In this case, the studied groups referred to the previous specimens identifications. The discriminatory power of characteristics is represented by Wilks' *lambda* values.

The PCA analysis yielded three components accounted for 53.11% of the total variance of data set. The first axis explained 31.53%, the second 12.14% and the third 9.43%. The strongest correlation ($r > 0.60$) with the first axis showed eight features. The first axis was mostly influenced by variables connected with reproductive organs such as length of hypanthium, petals, filaments, anthers, style, and sepals. The second axis was mainly influenced by two ratios: style/filament length and style/petals length. Additionally, two characters referring to the dimensions of bracts showed factor loadings closely to the assumed limit of significance.

The DA showed that thirteen of the studied characters are statistically significant (Wilks' *lambda* 0.889128, $F(48.424) = 20.79059$, $p < 0.0000$), including traits referring to vegetative and generative organs. The results of the chi-square test for all canonical roots proved their statistical significance.

The results of the chi-square test for all canonical roots proved their statistical significance. The first root was mostly influenced by the length of hypanthium, petals, filaments, anthers, sepals and sepal tips length, and by two ratio values: style/ sepals length and sepal tips/ sepals length. The width and length of lower leaves and bracts, length of filaments, sepals, sepal tips, as well as three ratios: lower leaves length/ width, anthers/ filaments length, and sepal tips/ sepals length turned out to be the most important for the separation of specimens along the second axis.

In order to compare the ranges of variability of the 3 species depending on latitude Europe was divided to 3 parts: Z. Northern Europe encompassed locations in the Scandinavian countries, as well as Lithuania, Latvia, Estonia, and selected sites in Russia. Southern Europe included sites in Italy, Portugal, and certain regions of France and Russia. All remaining locations are classified as part of Central Europe. Analyses of intraspecific diversity within three species showed no significant differences in qualitative or quantitative characteristics based on latitude.
